# Implementation of arbitrary polyhedral elements for automatic dynamic analyses of three-dimensional structures

**DOI:** 10.1038/s41598-022-07996-6

**Published:** 2022-03-09

**Authors:** Lei Zhou, Jianbo Li, Gao Lin

**Affiliations:** 1 State Key Laboratory of Coastal and Offshore Engineering, Dalian University of Technology, 2 Linggong Road, Dalian, 116024 China; 2 Institute of Earthquake Engineering, Faculty of Infrastructure Engineering, Dalian University of Technology, 2 Linggong Road, Dalian, 116024 China

**Keywords:** Engineering, Civil engineering

## Abstract

The transition from the geometry to the mesh can be rather difficult, manual and time-consuming, especially for the large scale complex structures. The procedure of mesh generation needs massive human intervention making the automatic engineering analyses of structures from CAD geometry models hardly possible. This paper focuses on implementing a polyhedron element with arbitrary convex topology based on the Scaled Boundary Finite Element Method (SBFEM) in ABAQUS on the strength of the interface of UEL (the subroutine to define a general user-defined element) for automatic dynamic analyses of three-dimensional structures. This implementation empowers ABAQUS to analyze any model with arbitrary polyhedron elements. When the geometry of a structure is obtained from CAD, the dynamic analyses can be launched seamlessly and automatically. Cases of a cantilever subjected to a dynamic harmonic excitation with the traditional hexahedron element and this polyhedron element are compared to verify the accuracy of the UEL. Taking a practical example of the Soil-Structure Interaction analysis of a Nuclear Power Plant, the applicability and performance of this implementation are tested. The results of the two examples confirm that this polyhedron element based on SBFEM can be more accurate using much less degrees of freedom and its implementation in ABAQUS is robust and compatible.

## Introduction

As the development of the Computer Aided Design (CAD) and Computer Aided Engineering (CAE) tools in the industry, the design, safety assessment, health monitoring of some important modern infrastructures tend to need more and more refined and sophisticated geometry models and mesh models. Nevertheless, there is a gap between the CAD and the CAE holding a barrier between the geometry model for design and the mesh model for engineering analysis. Because except for some rarely used meshless methods, it is a difficult, manual and time-consuming process to generate a fine mesh from a complex geometry. During the manual mesh generation, the balance of accuracy and efficiency should be examined, especially for dynamic analysis in the time domain. A basic requirement is to assign heterogeneous distributed grid densities for the different location of the geometry model to balance the calculation accuracy and efficiency, which are case-sensitive, completely experimental and can be rather tedious. The automatic mesh generation technique with polyhedron elements can break this barrier due to the flexible topology that makes them highly adaptable to the complex shape and smooth transition of mesh density, which is the foundation of the automatic engineering analysis.

Since the arising of numerical methods for the Partial Differential Equation (PDE), the mesh generation has become a primary procedure for these methods that need spatial discretization like the Finite Element Method (FEM) and the Finite Difference Method (FDM). From a geometrical point of view, a mesh is the approximate shape representation of the domains or the media (or can be collectively referred to as configurations) of the original physical problem, which consists of many non-overlapping continuous small domains called elements. These elements have certain topology defined by geometric feature points such as vertices called nodes, where the degrees of freedom are defined on. Except that a numerical analysis cannot be carried on without a mesh, the quality, size and type of the mesh have a great influence on the reliability of the results as well.

For the last four decades, lots of mesh generation technologies have been developed from two-dimension (2D) domain to three-dimension (3D) domain on the strength of the computer graphics. During the 1950s, mesh generation techniques based on coordinate transformations firstly came up, such as the conformal mapping^1,2^ technique. This technique was extended to simple 3D configurations by South et al.^3^ and Baker^4^. Although the methods above can generate structured hexahedral or quadrilateral meshes with high quality, the process of these methods requires lots of time, manual guidance and very specific geometry. In the 1980s, triangulation methods emerged generating unstructured triangular meshes for 2D configurations or tetrahedral meshes for 3D configurations. After the generation of a triangulation mesh, an unstructured hexahedral mesh can be obtained by the domain partition^5–8^. Unstructured meshes can always be created by triangulation methods for any intricate geometry automatically and efficiently. But these kinds of meshes have low accuracy and additional artificial stiffness, especially the first order ones. On the other hand, for a same domain the degrees of freedom of a triangulation mesh are much more than those of a hexahedral or quadrilateral mesh. If the mesh requirement of conforming the boundary is omitted, the Cartesian methods can automatically generate octree meshes that contain particular polyhedron elements without creating surface meshes. But the mesh created by this method has a poor accuracy on boundary vicinity leading to an inaccurate boundary condition. At the moment, for complex geometries only the polyhedron mesh generation technique can be fully automatic with few human interventions.

Polyhedron meshes such as octree meshes have been widely used in fluid mechanics exhibiting many topological properties that make them highly and automatically adaptable to the complex shape and smooth transition of mesh density^9,10^. Nevertheless, the application of polyhedron elements for continuum mechanics is few because the topology of a polyhedron is inconstant. Hence it is impossible to construct analytic shape functions for arbitrary polyhedron elements with varying topologies. Some kind of interpolation schemes must be applied. Two kinds of interpolation schemes were developed. The first one was based on natural neighbor interpolations proposed by Sibson^11,12^. Traversoni^13^ and Sambridge et al.^14^ introduced Sibson interpolations modified by Watson^15^ to the Galerkin-type natural neighbor, which was extended to continuum by Sukumar et al.^16,17^. Belikov et al. raised the Laplacian and non-Sibson interpolants^18,19^ and the polygonal finite element interpolants^20,21^. The other one was built on generalized barycentric coordinates, which was brought up by Wachspress^22^. This kind of interpolants was further developed by Warren^23,24^, Meyer et al.^25^, Floater et al.^26,27^, Lipman^28^, and Warren et al.^29^. However, the interpolation scheme generally needs considerable calculation to get adequate accuracy.

Another approach to constructing polyhedral elements is to utilize SBFEM. In mid-1990s, SBFEM also known as the consistent infinitesimal finite-element-cell method was bought up by Wolf and Song as an extension to FEM to simulate the wave propagation in the unbounded domain^30–33^. As a similarity-based semi-analytical numerical approach for PDE, SBFEM has many advantages over the traditional FEM and Boundary Element Method (BEM) exhibiting high accuracy and flexibility^34,35^. SBFEM just needs boundary discretization reducing the problem spatial dimension by one with no need for a fundamental solution and the solution along the radial direction is in a precise closed-form. For the unbounded media, SBFEM can perfectly satisfy the boundary condition of the radiation damping. The unit-impulse response matrix in the time domain and the dynamic-stiffness matrix in the frequency domain of the unbounded media can be directly obtained, which is equivalent to a time and space coupling artificial non-reflecting boundary^36^. For the bounded media, the SBFEM is derived in a wedge media. The wedge media can be assembled into an arbitrary convex polyhedron (3D) or polygon (2D) super element revolving around the similarity center. If this assembly is not occlusive and the crack tip is positioned in the similarity center, the singularity of the field function can be directly described by the analytical solution along the radical crack surface. This superiority of mesh adaptability can be combined with the automatic mesh generation technique based on polyhedron elements.

The first try was a SBFEM formulation for arbitrary polyhedral elements and a simple method to generate polyhedral meshes based on octree presented by Talebi et al.^37^. To automatically generate an octree polyhedral mesh from the Standard Tessellation Language (STL) widely used in the 3D printing and Computer Aided Design (CAD), Liu et al.^38^ came up with a two-steps method. The first step is to generate an octree mesh within the domain enclosed by the surface defined in the STL. The second step is to cut the polyhedral elements with the surface. After the refinement of the second step, the poor boundary accuracy of the octree mesh is improved. Furthermore, Zhang et al.^39^ applied the SBFEM polyhedral elements to the non-matching meshes by adding nodes to the elements adjacent to the interface. By now, there is a slight limitation when using SBFEM polyhedral elements. Since the boundary is discretized by FEM elements, the facets of a polyhedral element must be a triangle or quadrilateral, which may lead to a facet division process. To resolve this limitation of SBFEM, Ooi et al.^40^ derived a dual SBFEM formulation over arbitrary faceted star convex polyhedron. On the other hand, Zou et al.^41^ brought up a FEM polyhedral element formulation with shape functions derived from SBFEM circumventing this limitation as well. The automatic polyhedral element based on SBFEM was applied to dynamic nonlinear analysis of hydraulic engineering^42^, concrete fracture modelling^43^, damage modelling^44,45^, Soil-Structure Interaction (SSI) analysis of NPP^46^, etc. These applications of SBFEM polyhedral meshes were mostly implemented by self-developed programs. Few of them integrate the SBFEM into the universal FEM software such as ABAQUS^43,47^. And these integrations is suitable for traditional FEM elements with fixed topologies only. The integration of SBFEM and the universal FEM software can promote each other. Implementation of arbitrary polyhedral elements in ABAQUS is still in need.

In this paper, arbitrary polyhedron elements based on SBFEM for three dimensional continuum is developed and implemented in ABAQUS facilitating automatic dynamic analyses of structures. The governing equation of the bounded 3D elastic continuum in a polyhedron domain is derived from the SBFEM in “SBFEM governing equation for the elastic continuum” section. The FEM-coupled characteristic matrix of a polyhedron element can be obtained from the solution of the governing equation. The topology feature and corresponding data structure of a polyhedron is analyzed in “Topology and data structure of an arbitrary polyhedron element” section. The user element subroutine of UEL is developed associated with the Hilber-Hughes-Taylor (HHT) implicit time integration scheme in “Implementation in ABAQUS’ section. In “Numerical application” section, there are two examples for the UEL. A cantilever subjected to a harmonic excitation with the traditional hexahedron element and the SBFEM-based polyhedron element are compared to confirm the accuracy of the UEL. Then taking a practical example of the soil-structure dynamic interaction analysis of a nuclear power plant, the applicability and performance of this implementation of polyhedron elements is tested.

## SBFEM governing equation for the elastic continuum

**Figure 1:**
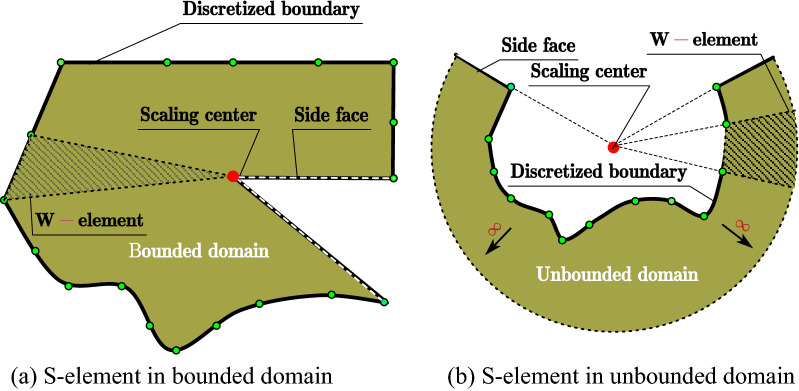
Spatial discretization of SBFEM for (**a**) S-element in bounded domain, (**b**) S-element in unbounded domain.

**Figure 2:**
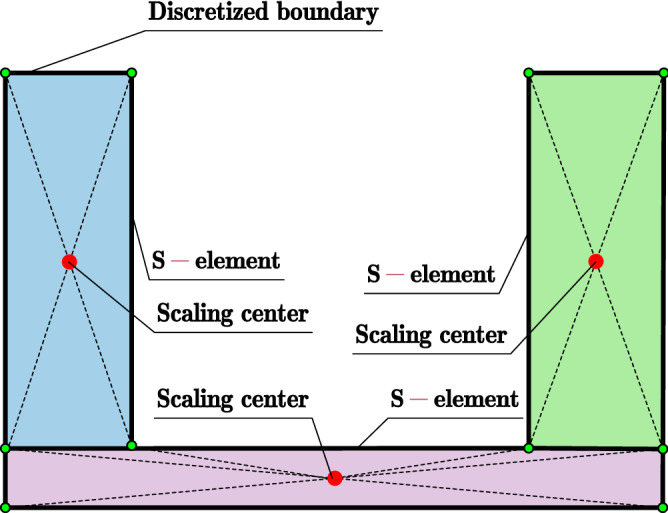
Bounded domain divided by three S-elements.

SBFEM is a numerical method for solving PDE. The PDE is transformed into ordinary differential equations after specific spatial discretization, which is different from FEM, BEM, etc. SBFEM needs boundary discretization only decreasing the spatial dimension by 1 and remains analytical in the radial direction without the need of a foundation solution. The derivation of the SBFEM governing equation of elastic continuum is based on a wedge area defined as W-element. The governing equations of W-elements that shared the same scaling center can be assembled by degrees of freedom of adjacent W-elements (see Fig. 1) leading to a piece-wise boundary of the discretized domain defined as an S-element. The choice of the scaling center location can be pretty casual. The boundary of the discretized domain must be visible from the scaling center is the only geometry requirement that needs to be satisfied. The bounded domain can be divided into multiple S-elements to meet the requirement of the scaling center or to improve the poor geometry of W-elements (see Fig. 2). Apparently, the geometry of the S-element can be rather flexible for any convex polyhedron with only triangle or quadrilateral facets.

The detailed derivation of SBFEM governing equation is well documented in many literatures^33,48–52^. For the sake of integrity, only key equations are given below. The equations in this section are given in linear algebraic notation for the convenience of reading and programming. The brace symbol of $\left\{ \cdot \right\}$ denotes a matrix and the bracket symbol of $\left[ \cdot \right]$ denotes a column vector.

### Partial differential equations of dynamic elastic mechanics

According to the theory of continuum mechanics, three basic equations are derived as

1 $$\left[ A \right]\left\{ \sigma \right\} + \left\{ f \right\} = \left\{ 0 \right\},$$

2 $$\left\{ \varepsilon \right\} = \left[ L \right]\left\{ u \right\},$$

3 $$\left\{ \sigma \right\} = \left[ D \right]\left\{ \varepsilon \right\},$$

where $\left[ A \right]$ denotes the spatial partial derivative operator, $\left\{ \sigma \right\}$ denotes the stress vector, $\left\{ \varepsilon \right\}$ denotes the strain vector, $\left\{ f \right\}$ denotes the body forces that include the inertial force for the dynamic analysis. The detailed definitions of them are

4 $$\left[ A \right] = \left[ {\begin{array}{*{20}c} {\frac{\partial }{\partial x}} & 0 & 0 & {\frac{\partial }{\partial y}} & 0 & {\frac{\partial }{\partial z}} \\ 0 & {\frac{\partial }{\partial y}} & 0 & {\frac{\partial }{\partial x}} & {\frac{\partial }{\partial z}} & 0 \\ 0 & 0 & {\frac{\partial }{\partial z}} & 0 & {\frac{\partial }{\partial y}} & {\frac{\partial }{\partial x}} \\ \end{array} } \right],$$

5 $$\left\{ {\sigma \left( {x, y, z} \right)} \right\} = \left\{ {\begin{array}{*{20}c} {\sigma_{x} } \\ {\sigma_{y} } \\ {\sigma_{z} } \\ {\tau_{xy} } \\ {\tau_{yz} } \\ {\tau_{zx} } \\ \end{array} } \right\},$$

6 $$\left\{ {f\left( {x, y, z} \right)} \right\} = \left\{ {\begin{array}{*{20}c} {f_{x} \left( {x, y, z} \right)} \\ {f_{y} \left( {x, y, z} \right)} \\ {f_{z} \left( {x, y, z} \right)} \\ \end{array} } \right\} = \left\{ {p\left( {x, y, z} \right)} \right\} - \rho \left\{ {\ddot{u}\left( {x, y, z} \right)} \right\}.$$

The equilibrium equations (Eq. (1)), the geometric equations (Eq. (2)) and the constitutive equations (Eq. (3)) can be combined into one set of partial differential equations of 3D dynamic elastic mechanics with the displacement vector filed as the unknown variable as

7 $$\begin{aligned} & \left[ L \right]^{{\text{T}}} \left[ D \right]\left[ L \right]\left\{ u \right\} + \left\{ p \right\} - \rho \left\{ {\ddot{u}} \right\} = \left\{ 0 \right\}, \\ & \left[ L \right] = \left[ A \right]^{{\text{T}}} = \left[ {\begin{array}{*{20}c} {\frac{\partial }{\partial x}} & 0 & 0 \\ 0 & {\frac{\partial }{\partial y}} & 0 \\ 0 & 0 & {\frac{\partial }{\partial z}} \\ {\frac{\partial }{\partial y}} & {\frac{\partial }{\partial x}} & 0 \\ 0 & {\frac{\partial }{\partial z}} & {\frac{\partial }{\partial y}} \\ {\frac{\partial }{\partial z}} & 0 & {\frac{\partial }{\partial x}} \\ \end{array} } \right], \\ \end{aligned}$$

where $$\left\{ {u\left( {x, y, z} \right)} \right\} = \left[ {\begin{array}{*{20}c} {u_{x} \left( {x, y, z} \right)} & {u_{y} \left( {x, y, z} \right)} & {u_{z} \left( {x, y, z} \right)} \\ \end{array} } \right]^{{\text{T}}}$$ denotes the displacement vector filed, $\left[ D \right]$ denotes the material property matrix, $\left\{ p \right\}$ denotes the body forces except the d'Alembert inertial force, $\rho$ denotes the density, $\left[ L \right]$ denotes the spatial partial derivative operator.

The linear elastic material property matrix $\left[ D \right]$ is given by

8 $$\begin{aligned} \left[ D \right] & = \left[ {\begin{array}{*{20}c} {\lambda + 2G} & \lambda & \lambda & 0 & 0 & 0 \\ \lambda & {\lambda + 2G} & \lambda & 0 & 0 & 0 \\ \lambda & \lambda & {\lambda + 2G} & 0 & 0 & 0 \\ 0 & 0 & 0 & G & 0 & 0 \\ 0 & 0 & 0 & 0 & G & 0 \\ 0 & 0 & 0 & 0 & 0 & G \\ \end{array} } \right], \\ \lambda & = \frac{E\nu }{{\left( {1 + \nu } \right)\left( {1 - 2\nu } \right)}},\quad G = \frac{E}{{2\left( {1 + \nu } \right)}}, \\ \end{aligned}$$

where $\lambda$ denotes the Lame parameter, $E$ denotes the Young’s modulus, $G$ denotes the shear modulus, $\nu$ denotes the Poisson’s ratio.

Equation (7) represents a set of second order linear nonhomogeneous partial differential equations with constant coefficients in Cartesian coordinate, namely Navier equations. One way to solve the Navier equations analytically is to transform them into wave equations of potential functions by Helmholtz’s theorem. The other way is to solve the Navier equations numerically by spatial and time discretization.

Two kinds of boundary conditions and one initial condition should be introduced to get the definite solution of the fundamental equation Eq. (7). One boundary condition is the geometry boundary condition on the surface $S_{u}$ defined as

9 $$\left\{ u \right\} = \left\{ {\overline{u}} \right\}\;\;on\;\;S_{u} ,$$

where $\left\{ {\overline{u}} \right\}$ is the constant displacement on the surface of $S_{u}.$

The other one is the surface traction boundary condition on the surface $S_{\sigma }$ defined as

10 $$\left[ n \right]\left\{ \sigma \right\} = \left\{ {\overline{T}} \right\}\;\;on\;\;S_{\sigma } ,$$

11 $$\left\{ {\overline{T}\left( {x, y, z} \right)} \right\} = \left\{ {\begin{array}{*{20}c} {\overline{T}_{x} } \\ {\overline{T}_{y} } \\ {\overline{T}_{z} } \\ \end{array} } \right\},$$

12 $$\left[ {n\left( {x, y, z} \right)} \right] = \left[ {\begin{array}{*{20}c} {n_{x} } & 0 & 0 & {n_{y} } & 0 & {n_{z} } \\ 0 & {n_{y} } & 0 & {n_{x} } & {n_{z} } & 0 \\ 0 & 0 & {n_{z} } & 0 & {n_{y} } & {n_{x} } \\ \end{array} } \right],$$

where $\left\{ {\overline{T}} \right\}$ is the constant surface traction force per area on $S_{\sigma }$, $\left[ n \right]$ is the orientation cosine matrix, $n_{x} \left( {x, y, z} \right)$, $n_{y} \left( {x, y, z} \right)$, and $n_{z} \left( {x, y, z} \right)$ are the orientation cosine of the outer normal vector of the surface $S_{\sigma }$.

The initial condition can be defined as

13 $$\left\{ {u\left( {x, y, z, t} \right)} \right\} = \left\{ {\overline{u}\left( {x, y, z} \right)} \right\}\;\;when\;\;t = 0,$$

14 $$\left\{ {\dot{u}\left( {x, y, z, t} \right)} \right\} = \left\{ {\overline{{\dot{u}}} \left( {x, y, z} \right)} \right\}\;\;when\;\;t = 0,$$

where $\left\{ {\overline{u}} \right\}$ is the constant displacement of the domain when $t = 0$, $\left\{ {\overline{{\dot{u}}} } \right\}$ is the constant velocity of the domain when $t = 0$.

### Coordinate mapping based on scaled boundary transformation

**Figure 3:**
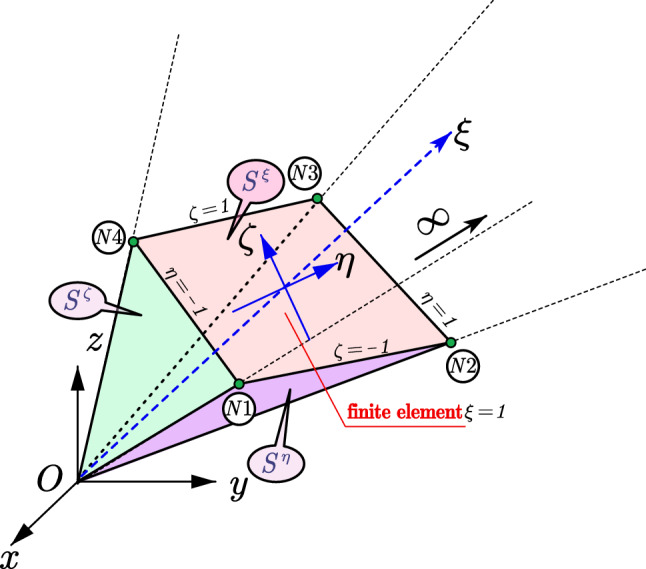
Spatial discretization of the W-element.

**Figure 4:**
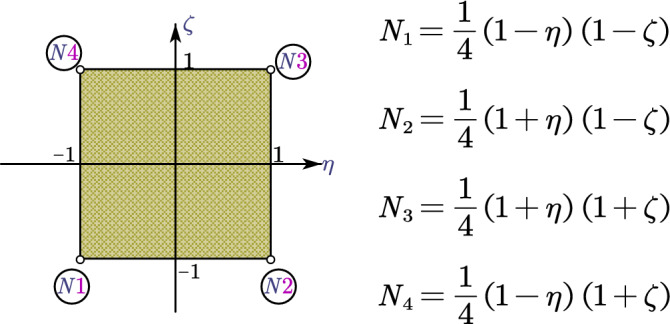
Four nodes linear plane finite element.

Taking one W-element as an example (see Fig. 3), the boundary is discretized with one plane four nodes linear finite element with circumferential axis of $\eta$ and $\zeta$ (see Fig. 4). Other 2D finite elements such as the quadratic element with eight nodes can be used for this boundary discretization as well^51^.

The origin of the global Cartesian coordinate system of the nodes needs to be translated to the scaling center by

15 $$\begin{aligned} \left\{ {x_{e} } \right\} & = \left\{ {\tilde{x}_{e} } \right\} - \tilde{x}_{e}^{0} , \\ \left\{ {y_{e} } \right\} & = \left\{ {\tilde{y}_{e} } \right\} - \tilde{y}_{e}^{0} , \\ \left\{ {z_{e} } \right\} & = \left\{ {\tilde{z}_{e} } \right\} - \tilde{z}_{e}^{0} , \\ \end{aligned}$$

where $\left\{ {\tilde{x}_{e} } \right\}$, $\left\{ {\tilde{y}_{e} } \right\}$, and $\left\{ {\tilde{z}_{e} } \right\}$ denote the global coordinates of the nodes, $\tilde{x}_{e}^{0}$, $\tilde{y}_{e}^{0}$, and $\tilde{z}_{e}^{0}$ denote the coordinates of the scaling center in global Cartesian coordinate system, $\left\{ {x_{e} } \right\}$, $\left\{ {y_{e} } \right\}$, and $\left\{ {z_{e} } \right\}$ denote the translated global coordinates of the nodes.

The mapping function between the global coordinates and the local coordinates of the points on the boundary is given by

16 $$\begin{aligned} \hat{x}\left( {\eta , \zeta } \right) & = \left\{ {N\left( {\eta ,{ } \zeta } \right)} \right\}^{{\text{T}}} \left\{ {x_{e} } \right\}, \\ \hat{y}\left( {\eta , \zeta } \right) & = \left\{ {N\left( {\eta , \zeta } \right)} \right\}^{{\text{T}}} \left\{ {y_{e} } \right\}, \\ \hat{z}\left( {\eta , \zeta } \right) & = \left\{ {N\left( {\eta , \zeta } \right)} \right\}^{{\text{T}}} \left\{ {z_{e} } \right\}, \\ \end{aligned}$$

where $$\left\{ {N\left( {\eta , \zeta } \right)} \right\}^{T} = \left[ {\begin{array}{*{20}c} {N_{1} } & {N_{2} } & {N_{3} } & {N_{4} } \\ \end{array} } \right]$$ denotes the shape function of the plane finite element, $\left\{ {x_{e} } \right\}$, $\left\{ {y_{e} } \right\}$, and $\left\{ {z_{e} } \right\}$ denote the global coordinates of the nodes that have been translated by Eq. (15), $\hat{x}\left( {\eta , \zeta } \right)$, $\hat{y}\left( {\eta ,{ } \zeta } \right)$, and $\hat{z}\left( {\eta , \zeta } \right)$ denote the global coordinates of arbitrary point on the boundary determined by the local coordinates $\left( {\eta , \zeta } \right)$.

The new local scale boundary coordinate is built for this W-element with the origin at the scaling center. Points on the plane finite element is scaled along the radical direction from the scaling center to the boundary, which is defined as the $\xi$ axis of the local scale boundary coordinate. The mapping function between the global coordinates and the local coordinates of the points in the W-element is given by

17 $$\begin{array}{*{20}c} {x\left( {\xi , \eta , \zeta } \right) = \xi \hat{x} = \xi \left\{ {N\left( {\eta , \zeta } \right)} \right\}^{{\text{T}}} \left\{ {x_{e} } \right\},} \\ {y\left( {\xi , \eta , \zeta } \right) = \xi \hat{y} = \xi \left\{ {N\left( {\eta , \zeta } \right)} \right\}^{{\text{T}}} \left\{ {y_{e} } \right\},} \\ {z\left( {\xi , \eta , \zeta } \right) = \xi \hat{z} = \xi \left\{ {N\left( {\eta , \zeta } \right)} \right\}^{{\text{T}}} \left\{ {z_{e} } \right\},} \\ \end{array}$$

where $x\left( {\xi , \eta , \zeta } \right)$, $y\left( {\xi , \eta , \zeta } \right)$, and $z\left( {\xi , \eta ,{ } \zeta } \right)$ denote the local coordinates of the points in the W-element.

Equation (17) provides a mapping relation between the local and global coordinates. Based on the derivative method for compound function, the corresponding relationship of the derivative operators between two coordinates is given by

18 $$\left\{ {\begin{array}{*{20}c} {\frac{\partial }{\partial x}} \\ {\frac{\partial }{\partial y}} \\ {\frac{\partial }{\partial z}} \\ \end{array} } \right\} = \left[ J \right]^{ - 1} \left\{ {\begin{array}{*{20}c} {\frac{\partial }{\partial \xi }} \\ {\frac{\partial }{\partial \eta }} \\ {\frac{\partial }{\partial \zeta }} \\ \end{array} } \right\},$$

where $\left[ J \right]$ is the Jacobian matrix expressed as

19 $$\left[ J \right] = \left[ {\begin{array}{*{20}c} {\frac{\partial x}{{\partial \xi }}} & {\frac{\partial y}{{\partial \xi }}} & {\frac{\partial z}{{\partial \xi }}} \\ {\frac{\partial x}{{\partial \eta }}} & {\frac{\partial y}{{\partial \eta }}} & {\frac{\partial z}{{\partial \eta }}} \\ {\frac{\partial x}{{\partial \zeta }}} & {\frac{\partial y}{{\partial \zeta }}} & {\frac{\partial z}{{\partial \zeta }}} \\ \end{array} } \right].$$

The variable $\xi$ can be isolated from Eq. (19) as

20 $$\left[ {J\left( {\xi ,\eta ,\zeta } \right)} \right] = \left[ {\begin{array}{*{20}c} 1 & 0 & 0 \\ 0 & \xi & 0 \\ 0 & 0 & \xi \\ \end{array} } \right]\left[ {\hat{J}} \right],$$

with the Jacobian matrix on the boundary $\left[ {\hat{J}} \right]$ defined as

21 $$\left[ {\hat{J}\left( {\eta ,\zeta } \right)} \right] = \left[ {\begin{array}{*{20}c} {\hat{x}} & {\hat{y}} & {\hat{z}} \\ {\hat{x}_{,\eta } } & {\hat{y}_{,\eta } } & {\hat{z}_{,\eta } } \\ {\hat{x}_{,\zeta } } & {\hat{y}_{,\zeta } } & {\hat{z}_{,\zeta } } \\ \end{array} } \right],$$

where $\left( \cdot \right)_{,\eta }$ denotes differentiating with respect to $\eta$, $\left( \cdot \right)_{,\zeta }$ denotes differentiating with respect to $\zeta$.

Based on the space analytical geometric theory, the geometry properties in the local coordinate system can be derived. The infinitesimal line vectors in the axis direction are given by

22 $$\overrightarrow {d\xi } = d\xi \left( {\hat{x}, \hat{y},{ } \hat{z}} \right),\quad { }\overrightarrow {d\eta } = \xi d\eta \left( {\hat{x}_{,\eta } , \hat{y}_{,\eta } , \hat{z}_{,\eta } } \right),\quad \overrightarrow {d\zeta } = \xi d\zeta \left( {\hat{x}_{,\zeta } , \hat{y}_{,\zeta } ,{ } \hat{z}_{,\zeta } } \right),$$

where $\left( \cdot \right)$ denotes a vector.

The infinitesimal volume is given by

23 $$dV = \overrightarrow {d\xi } \cdot \overrightarrow {d\eta } \times \overrightarrow {d\zeta } = \xi^{2} \left| {\left[ {\hat{J}} \right]} \right|d\xi d\eta d\zeta ,$$

where $\left| \cdot \right|$ denotes the determinant of the matrix.

The normal vectors of the coordinate surfaces are given by

24 $$\begin{aligned} \overrightarrow {{g^{\xi } }} & = \left( {\hat{y}_{,\eta } \hat{z}_{,\zeta } - \hat{y}_{,\zeta } \hat{z}_{,\eta } , \hat{z}_{,\eta } \hat{x}_{,\zeta } - \hat{z}_{,\zeta } \hat{x}_{,\eta } , \hat{x}_{,\eta } \hat{y}_{,\zeta } - \hat{x}_{,\zeta } \hat{y}_{,\eta } } \right), \\ \overrightarrow {{g^{\eta } }} & = \left( {\hat{y}_{,\zeta } \hat{z} - \hat{y}\hat{z}_{,\zeta } , \hat{z}_{,\zeta } \hat{x} - \hat{z}\hat{x}_{,\zeta } , \hat{x}_{,\zeta } \hat{y} - \hat{x}\hat{y}_{,\zeta } } \right), \\ \overrightarrow {{g^{\zeta } }} & = \left( {\hat{y}\hat{z}_{,\eta } - \hat{y}_{,\eta } \hat{z},{ } \hat{z}\hat{x}_{,\eta } - \hat{z}_{,\eta } \hat{x}, \hat{x}\hat{y}_{,\eta } - \hat{x}_{,\eta } \hat{y}} \right), \\ \end{aligned}$$

where $\overrightarrow {{g^{\xi } }}$, $\overrightarrow {{g^{\eta } }}$, and $\overrightarrow {{g^{\zeta } }}$ denote the normal vector of the coordinate surface of $S^{\xi }$, $S^{\eta }$ and $S^{\zeta }$.

The length of the normal vectors $g^{\xi }$, $g^{\eta }$, and $g^{\zeta }$ and the unit normal vectors $\overrightarrow {{n^{\xi } }}$, $\overrightarrow {{n^{\eta } }}$, and $\overrightarrow {{n^{\zeta } }}$ are defined as

25 $$g^{\xi } = \left| {\overrightarrow {{g^{\xi } }} } \right|,\quad g^{\eta } = \left| {\overrightarrow {{g^{\eta } }} } \right|,\quad g^{\zeta } = \left| {\overrightarrow {{g^{\zeta } }} } \right|,$$

26 $$\overrightarrow {{n^{\xi } }} = \frac{{\overrightarrow {{g^{\xi } }} }}{{g^{\xi } }} = \left( {n_{x}^{\xi } , n_{y}^{\xi } , n_{z}^{\xi } } \right),\quad \overrightarrow {{n^{\eta } }} = \frac{{\overrightarrow {{g^{\eta } }} }}{{g^{\eta } }} = \left( {n_{x}^{\eta } , n_{y}^{\eta } , n_{z}^{\eta } } \right),\quad \overrightarrow {{n^{\zeta } }} = \frac{{\overrightarrow {{g^{\zeta } }} }}{{g^{\zeta } }} = \left( {n_{x}^{\zeta } , n_{y}^{\zeta } , n_{z}^{\zeta } } \right),$$

where $\left| \cdot \right|$ denotes the length of the vector.

The infinitesimal area of the coordinate surfaces is given by

27 $$\begin{aligned} dS^{\xi } & = \left| {\overrightarrow {d\eta } \times \overrightarrow {d\zeta } } \right| = \xi^{2} g^{\xi } d\eta d\zeta , \\ dS^{\eta } & = \left| {\overrightarrow {d\zeta } \times \overrightarrow {d\xi } } \right| = \xi g^{\eta } d\xi d\zeta , \\ dS^{\zeta } & = \left| {\overrightarrow {d\xi } \times \overrightarrow {d\eta } } \right| = \xi g^{\zeta } d\xi d\eta . \\ \end{aligned}$$

$\left[ {\hat{J}} \right]^{ - 1}$ can be expressed as

28 $$\left[ {\hat{J}} \right]^{ - 1} = \frac{1}{{\left| {\left[ {\hat{J}} \right]} \right|}}\left[ {\begin{array}{*{20}c} {g^{\xi } n_{x}^{\xi } } & {g^{\eta } n_{x}^{\eta } } & {g^{\zeta } n_{x}^{\zeta } } \\ {g^{\xi } n_{y}^{\xi } } & {g^{\eta } n_{y}^{\eta } } & {g^{\zeta } n_{y}^{\zeta } } \\ {g^{\xi } n_{z}^{\xi } } & {g^{\eta } n_{z}^{\eta } } & {g^{\zeta } n_{z}^{\zeta } } \\ \end{array} } \right].$$

Substituting Eq. (28) into Eq. (18), the derivative operators in the local coordinates is given by

29 $$\begin{aligned} \frac{\partial }{\partial x} & = \frac{{g^{\xi } }}{{\left| {\left[ {\hat{J}} \right]} \right|}}n_{x}^{\xi } \frac{\partial }{\partial \xi } + \frac{1}{\xi }\left( {\frac{{g^{\eta } }}{{\left| {\left[ {\hat{J}} \right]} \right|}}n_{x}^{\eta } \frac{\partial }{\partial \eta } + \frac{{g^{\zeta } }}{{\left| {\left[ {\hat{J}} \right]} \right|}}n_{x}^{\zeta } \frac{\partial }{\partial \zeta }} \right), \\ \frac{\partial }{\partial y} & = \frac{{g^{\xi } }}{{\left| {\left[ {\hat{J}} \right]} \right|}}n_{y}^{\xi } \frac{\partial }{\partial \xi } + \frac{1}{\xi }\left( {\frac{{g^{\eta } }}{{\left| {\left[ {\hat{J}} \right]} \right|}}n_{y}^{\eta } \frac{\partial }{\partial \eta } + \frac{{g^{\zeta } }}{{\left| {\left[ {\hat{J}} \right]} \right|}}n_{y}^{\zeta } \frac{\partial }{\partial \zeta }} \right), \\ \frac{\partial }{\partial z} & = \frac{{g^{\xi } }}{{\left| {\left[ {\hat{J}} \right]} \right|}}n_{z}^{\xi } \frac{\partial }{\partial \xi } + \frac{1}{\xi }\left( {\frac{{g^{\eta } }}{{\left| {\left[ {\hat{J}} \right]} \right|}}n_{z}^{\eta } \frac{\partial }{\partial \eta } + \frac{{g^{\zeta } }}{{\left| {\left[ {\hat{J}} \right]} \right|}}n_{z}^{\zeta } \frac{\partial }{\partial \zeta }} \right). \\ \end{aligned}$$

Substituting Eq. (29) into Eq. (7), the spatial partial derivative operator $\left[ L \right]$ in local coordinate system is given by

30 $$\left[ L \right] = \left[ {b^{1} } \right]\frac{\partial }{\partial \xi } + \frac{1}{\xi }\left( {\left[ {b^{2} } \right]\frac{\partial }{\partial \eta } + \left[ {b^{3} } \right]\frac{\partial }{\partial \zeta }} \right),$$

where $\left[ {b^{1} } \right]$, $\left[ {b^{2} } \right]$, and $\left[ {b^{3} } \right]$ are defined as

31 $$\left[ {b^{1} } \right] = \frac{{g^{\xi } }}{{\left| {\left[ {\hat{J}} \right]} \right|}}\left[ {\begin{array}{*{20}c} {n_{x}^{\xi } } & 0 & 0 \\ 0 & {n_{y}^{\xi } } & 0 \\ 0 & 0 & {n_{z}^{\xi } } \\ {n_{y}^{\xi } } & {n_{x}^{\xi } } & 0 \\ 0 & {n_{z}^{\xi } } & {n_{y}^{\xi } } \\ {n_{z}^{\xi } } & 0 & {n_{x}^{\xi } } \\ \end{array} } \right],$$

32 $$\left[ {b^{2} } \right] = \frac{{g^{\eta } }}{{\left| {\left[ {\hat{J}} \right]} \right|}}\left[ {\begin{array}{*{20}c} {n_{x}^{\eta } } & 0 & 0 \\ 0 & {n_{y}^{\eta } } & 0 \\ 0 & 0 & {n_{z}^{\eta } } \\ {n_{y}^{\eta } } & {n_{x}^{\eta } } & 0 \\ 0 & {n_{z}^{\eta } } & {n_{y}^{\eta } } \\ {n_{z}^{\eta } } & 0 & {n_{x}^{\eta } } \\ \end{array} } \right],$$

33 $$\left[ {b^{3} } \right] = \frac{{g^{\zeta } }}{{\left| {\left[ {\hat{J}} \right]} \right|}}\left[ {\begin{array}{*{20}c} {n_{x}^{\zeta } } & 0 & 0 \\ 0 & {n_{y}^{\zeta } } & 0 \\ 0 & 0 & {n_{z}^{\zeta } } \\ {n_{y}^{\zeta } } & {n_{x}^{\zeta } } & 0 \\ 0 & {n_{z}^{\zeta } } & {n_{y}^{\zeta } } \\ {n_{z}^{\zeta } } & 0 & {n_{x}^{\zeta } } \\ \end{array} } \right],$$

and they satisfy

34 $$\left( {\left| {\left[ {\hat{J}} \right]} \right|\left[ {b^{2} } \right]} \right)_{,\eta } + \left( {\left| {\left[ {\hat{J}} \right]} \right|\left[ {b^{3} } \right]} \right)_{,\zeta } = - 2\left| {\left[ {\hat{J}} \right]} \right|\left[ {b^{1} } \right].$$

Substituting Eq. (30) into Eqs. (2) and (3), the stress $\left\{ \sigma \right\}$ in local coordinate system is given by

35 $$\left\{ {\sigma \left( {\xi , \eta , \zeta } \right)} \right\} = \left[ D \right]\left( {\left[ {b^{1} } \right]\left\{ {u\left( {\xi , \eta , \zeta } \right)} \right\}_{,\xi } + \frac{1}{\xi }\left( {\left[ {b^{2} } \right]\left\{ {u\left( {\xi , \eta , \zeta } \right)} \right\}_{,\eta } + \left[ {b^{3} } \right]\left\{ {u\left( {\xi , \eta , \zeta } \right)} \right\}_{,\zeta } } \right)} \right).$$

Considering the surface traction boundary condition in Eq. (10) to Eq. (12), the surface traction on the coordinate surface is defined as

36 $$\left\{ {t^{\xi } } \right\} = \left\{ {\begin{array}{*{20}c} {t_{x}^{\xi } } & {t_{y}^{\xi } } & {t_{z}^{\xi } } \\ \end{array} } \right\}^{{\text{T}}} = \left[ {\begin{array}{*{20}c} {n_{x}^{\xi } } & 0 & 0 & {n_{y}^{\xi } } & 0 & {n_{z}^{\xi } } \\ 0 & {n_{y}^{\xi } } & 0 & {n_{x}^{\xi } } & {n_{z}^{\xi } } & 0 \\ 0 & 0 & {n_{z}^{\xi } } & 0 & {n_{y}^{\xi } } & {n_{x}^{\xi } } \\ \end{array} } \right]\left\{ {\begin{array}{*{20}c} {\sigma_{x} } \\ {\sigma_{y} } \\ {\sigma_{z} } \\ {\tau_{xy} } \\ {\tau_{yz} } \\ {\tau_{zx} } \\ \end{array} } \right\},$$

37 $$\left\{ {t^{\eta } } \right\} = \left\{ {\begin{array}{*{20}c} {t_{x}^{\eta } } & {t_{y}^{\eta } } & {t_{z}^{\eta } } \\ \end{array} } \right\}^{{\text{T}}} = \left[ {\begin{array}{*{20}c} {n_{x}^{\eta } } & 0 & 0 & {n_{y}^{\eta } } & 0 & {n_{z}^{\eta } } \\ 0 & {n_{y}^{\eta } } & 0 & {n_{x}^{\eta } } & {n_{z}^{\eta } } & 0 \\ 0 & 0 & {n_{z}^{\eta } } & 0 & {n_{y}^{\eta } } & {n_{x}^{\eta } } \\ \end{array} } \right]\left\{ {\begin{array}{*{20}c} {\sigma_{x} } \\ {\sigma_{y} } \\ {\sigma_{z} } \\ {\tau_{xy} } \\ {\tau_{yz} } \\ {\tau_{zx} } \\ \end{array} } \right\},$$

38 $$\left\{ {t^{\zeta } } \right\} = \left\{ {\begin{array}{*{20}c} {t_{x}^{\zeta } } & {t_{y}^{\zeta } } & {t_{z}^{\zeta } } \\ \end{array} } \right\}^{{\text{T}}} = \left[ {\begin{array}{*{20}c} {n_{x}^{\zeta } } & 0 & 0 & {n_{y}^{\zeta } } & 0 & {n_{z}^{\zeta } } \\ 0 & {n_{y}^{\zeta } } & 0 & {n_{x}^{\zeta } } & {n_{z}^{\zeta } } & 0 \\ 0 & 0 & {n_{z}^{\zeta } } & 0 & {n_{y}^{\zeta } } & {n_{x}^{\zeta } } \\ \end{array} } \right]\left\{ {\begin{array}{*{20}c} {\sigma_{x} } \\ {\sigma_{y} } \\ {\sigma_{z} } \\ {\tau_{xy} } \\ {\tau_{yz} } \\ {\tau_{zx} } \\ \end{array} } \right\},$$

where $\left\{ {t^{\xi } } \right\}$, $\left\{ {t^{\eta } } \right\}$, and $\left\{ {t^{\zeta } } \right\}$ denote the the surface traction on the coordinate surface of $\xi$, $\eta$, and $\zeta$ respectively.

Compared with Eq. (31) to Eqs. (33), (36) to Eq. (38) can be reformulated as

39 $$\left\{ {t^{\xi } } \right\} = \frac{{\left| {\left[ {\hat{J}} \right]} \right|}}{{g^{\xi } }}\left[ {b^{1} } \right]^{{\text{T}}} \left\{ \sigma \right\},$$

40 $$\left\{ {t^{\eta } } \right\} = \frac{{\left| {\left[ {\hat{J}} \right]} \right|}}{{g^{\eta } }}\left[ {b^{2} } \right]^{{\text{T}}} \left\{ \sigma \right\},$$

41 $$\left\{ {t^{\zeta } } \right\} = \frac{{\left| {\left[ {\hat{J}} \right]} \right|}}{{g^{\zeta } }}\left[ {b^{3} } \right]^{{\text{T}}} \left\{ \sigma \right\}.$$

### Spatial discretization of the displacement field on the boundary

**Figure 5:**
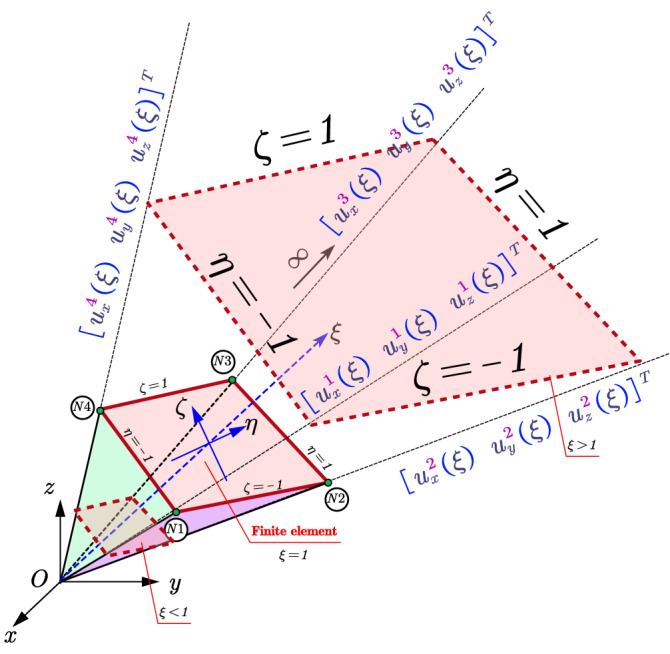
Displacement field discretization of the W-element.

Taking the same W-element in “Coordinate mapping based on scaled boundary transformation” section as example (see Fig. 5), the boundary is discretized with a finite element with circumferential axis of $\eta$ and $\zeta$ (see Fig. 4).

The displacement field on the boundary $\left\{ {u\left( {\eta , \zeta } \right)} \right\}$ can be expressed as

42 $$\left\{ {u\left( {\eta , \zeta } \right)} \right\} = \left[ {N\left( {\eta , \zeta } \right)} \right]\left\{ {u^{e} } \right\},$$

where $$\left[ {N\left( {\eta , \zeta } \right)} \right] = \left[ {\begin{array}{*{20}c} {N_{1} \left( {\eta , \zeta } \right)\left[ I \right]} & {N_{2} \left( {\eta , \zeta } \right)\left[ I \right]} & {N_{3} \left( {\eta , \zeta } \right)\left[ I \right]} & {N_{4} \left( {\eta , \zeta } \right)\left[ I \right]} \\ \end{array} } \right]$$ denotes the shape function of the finite element, $\left\{ {u^{e} } \right\}$ denotes the displacement values of the nodes, $\left[ I \right]$ denotes a $3 \times 3$ identity matrix.

The interpolation shown in Eq. (42) is applied to any coordinate surface of $S^{\xi }$. The displacement field $\left\{ {u\left( {\xi , \eta , \zeta } \right)} \right\}$ in the W-element can be expressed as

43 $$\left\{ {u\left( {\xi , \eta , \zeta } \right)} \right\} = \left[ {N\left( {\eta , \zeta } \right)} \right]\left\{ {u^{e} \left( \xi \right)} \right\},$$

where $\left\{ {u^{e} \left( \xi \right)} \right\}$ denotes the the displacement values along the rays starting from the scaling center to the nodes on the boundary.

Substituting Eq. (43) into Eq. (35), the discretization of the stress $\left\{ \sigma \right\}$ is given by

44 $$\left\{ \sigma \right\} = \left[ D \right]\left( {\left[ {B^{1} } \right]\left\{ {u^{e} \left( \xi \right)} \right\}_{,\xi } + \frac{1}{\xi }\left[ {B^{2} } \right]\left\{ {u^{e} \left( \xi \right)} \right\}} \right),$$

where $\left[ {B^{1} \left( {\eta , \zeta } \right)} \right]$, $\left[ {B^{2} \left( {\eta , \zeta } \right)} \right]$ are defined as

45 $$\left[ {B^{1} \left( {\eta , \zeta } \right)} \right] = \left[ {b^{1} } \right]\left[ N \right],$$

46 $$\left[ {B^{2} \left( {\eta , \zeta } \right)} \right] = \left[ {b^{2} } \right]\left[ N \right]_{,\eta } + \left[ {b^{3} } \right]\left[ N \right]_{,\zeta } .$$

### Equivalent integral equations and Galerkin weighted residual method

The SBFEM governing equation can be derived by the application of the weighted residual method or variation principle. In this section, the procedure of Galerkin weighted residual method is elaborated.

The equivalent integral form of Eq. (7) is given by

47 $$\begin{aligned} & \mathop \int \limits_{V} \left[ {w_{j} } \right]^{{\text{T}}} \left[ {b^{1} } \right]^{{\text{T}}} \left\{ \sigma \right\}_{,\xi } dV + \mathop \int \limits_{V} \left[ {w_{j} } \right]^{{\text{T}}} \frac{1}{\xi }\left( {\left[ {b^{2} } \right]^{{\text{T}}} \left\{ \sigma \right\}_{,\eta } + \left[ {b^{3} } \right]^{{\text{T}}} \left\{ \sigma \right\}_{,\zeta } } \right)dV \\ & \quad - \mathop \int \limits_{V} \left[ {w_{j} } \right]^{{\text{T}}} \rho \left\{ {\ddot{u}} \right\}dV + \mathop \int \limits_{V} \left[ {w_{j} } \right]^{{\text{T}}} \left\{ p \right\}dV = \left\{ 0 \right\}, \\ \end{aligned}$$

where $\left[ {w_{j} } \right]^{{\text{T}}}$ denotes the arbitrary weight function defined in Eq. (48), $\left[ \cdot \right]^{{\text{T}}}$ denotes the transpose of the matrix.

$\left[ {w_{j} } \right]^{{\text{T}}}$ is defined as

48 $$\left[ {w_{j} \left( {\xi ,\eta ,\zeta } \right)} \right] = w_{j} \left[ I \right] = w_{j} \left[ {\begin{array}{*{20}c} 1 & 0 & 0 \\ 0 & 1 & 0 \\ 0 & 0 & 1 \\ \end{array} } \right].$$

where $j = 1,{ }2,{ } \ldots ,m$ denotes the number of weight functions and $m \to \infty$.

Substituting Eq. (23) into Eq. (47) results in

49 $$\begin{aligned} & \overbrace {{\mathop \int \limits_{0}^{1} \xi^{2} \mathop \int \limits_{ - 1}^{1} \mathop \int \limits_{ - 1}^{1} \left[ {w_{j} } \right]^{{\text{T}}} \left[ {b^{1} } \right]^{{\text{T}}} \left\{ \sigma \right\}_{,\xi } \left| {\left[ {\hat{J}\left( {\eta , \zeta } \right)} \right]} \right|d\eta d\zeta d\xi }}^{{\text{I}}} \\ & \quad + \overbrace {{\mathop \int \limits_{0}^{1} \begin{array}{*{20}c} {\xi^{2} \mathop \int \limits_{ - 1}^{1} \mathop \int \limits_{ - 1}^{1} \left[ {w_{j} } \right]^{{\text{T}}} \frac{1}{\xi }\left( {\left[ {b^{2} } \right]^{{\text{T}}} \left\{ \sigma \right\}_{,\eta } + \left[ {b^{3} } \right]^{{\text{T}}} \left\{ \sigma \right\}_{,\zeta } } \right)\left| {\left[ {\hat{J}\left( {\eta , \zeta } \right)} \right]} \right|d\eta d\zeta d\xi } \\ \end{array} }}^{{{\text{II}}}} \\ & \quad + \overbrace {{\mathop \int \limits_{0}^{1} \xi^{2} \mathop \int \limits_{ - 1}^{1} \mathop \int \limits_{ - 1}^{1} \left[ {w_{j} } \right]^{{\text{T}}} \left\{ p \right\}\left| {\left[ {\hat{J}\left( {\eta , \zeta } \right)} \right]} \right|d\eta d\zeta d\xi }}^{{{\text{III}}}} \\ & \quad - \overbrace {{\mathop \int \limits_{0}^{1} \xi^{2} \mathop \int \limits_{ - 1}^{1} \mathop \int \limits_{ - 1}^{1} \left[ {w_{j} } \right]^{{\text{T}}} \rho \left\{ {\ddot{u}} \right\}\left| {\left[ {\hat{J}\left( {\eta , \zeta } \right)} \right]} \right|d\eta d\zeta d\xi }}^{{{\text{IV}}}} = \left\{ 0 \right\}. \\ \end{aligned}$$

Dealing the second term labeled II in Eq. (49) with Green's theorem results in

50 $$\begin{aligned} {\text{II}} & = \mathop \int \limits_{0}^{1} \begin{array}{*{20}c} {\xi \mathop \int \limits_{ - 1}^{1} \mathop \int \limits_{ - 1}^{1} \left[ {w_{j} } \right]^{{\text{T}}} \left( {\left[ {b^{2} } \right]^{{\text{T}}} \left\{ \sigma \right\}_{,\eta } + \left[ {b^{3} } \right]^{{\text{T}}} \left\{ \sigma \right\}_{,\zeta } } \right)\left| {\left[ {\hat{J}\left( {\eta , \zeta } \right)} \right]} \right|d\eta d\zeta d\xi } \\ \end{array} \\ { } & = \mathop \int \limits_{0}^{1} \begin{array}{*{20}c} {\xi \mathop {\iint }\limits_{{S^{\xi } }} \left[ {w_{j} } \right]^{{\text{T}}} \left( {\left[ {b^{2} } \right]^{{\text{T}}} \left\{ \sigma \right\}_{,\eta } + \left[ {b^{3} } \right]^{{\text{T}}} \left\{ \sigma \right\}_{,\zeta } } \right)\left| {\left[ {\hat{J}\left( {\eta , \zeta } \right)} \right]} \right|d\eta d\zeta d\xi } \\ \end{array} \\ & = \mathop \int \limits_{0}^{1} \begin{array}{*{20}c} {\xi \left( {\begin{array}{*{20}c} {\mathop {\oint }\limits_{{\Gamma^{\xi } }} \left[ {w_{j} } \right]^{{\text{T}}} \left( {\left( {\left| {\left[ {\hat{J}} \right]} \right|\left[ {b^{2} } \right]^{{\text{T}}} \left\{ \sigma \right\}} \right){\text{d}}\zeta + \left( {\left| {\left[ {\hat{J}} \right]} \right|\left[ {b^{3} } \right]^{{\text{T}}} \left\{ \sigma \right\}} \right){\text{d}}\eta } \right)} \\ { - \mathop {\iint }\limits_{{S^{\xi } }} \left( {\left( {\left( {\left[ {w_{j} } \right]^{{\text{T}}} \left| {\left[ {\hat{J}} \right]} \right|\left[ {b^{2} } \right]^{{\text{T}}} } \right)_{,\eta } + \left( {\left[ {w_{j} } \right]^{{\text{T}}} \left| {\left[ {\hat{J}} \right]} \right|\left[ {b^{3} } \right]^{{\text{T}}} } \right)_{,\zeta } } \right)\left\{ \sigma \right\}} \right){\text{d}}\eta {\text{d}}\zeta } \\ \end{array} } \right)d\xi ,} \\ \end{array} \\ \end{aligned}$$

where $\varGamma^{\xi }$ denotes the the boudnary of surafce $S^{\xi }$.

Substituting Eq. (39) to Eq. (41) into Eq. (50) results in

51 $$\begin{aligned} {\text{II}} & = \mathop \int \limits_{0}^{1} \begin{array}{*{20}c} {\xi \left( {\begin{array}{*{20}c} {\mathop {\oint }\limits_{{\Gamma^{\xi } }} \left[ {w_{j} } \right]^{{\text{T}}} \left( {\left| {\left[ {\hat{J}} \right]} \right|\left[ {b^{2} } \right]^{{\text{T}}} \left\{ \sigma \right\}{\text{d}}\zeta + \left| {\left[ {\hat{J}} \right]} \right|\left[ {b^{3} } \right]^{{\text{T}}} \left\{ \sigma \right\}{\text{d}}\eta } \right)} \\ { - \mathop {\iint }\limits_{{S^{\xi } }} \left( {\left( {\left[ {w_{j} } \right]^{{\text{T}}} \left| {\left[ {\hat{J}} \right]} \right|\left[ {b^{2} } \right]^{{\text{T}}} } \right)_{,\eta } + \left( {\left[ {w_{j} } \right]^{{\text{T}}} \left| {\left[ {\hat{J}} \right]} \right|\left[ {b^{3} } \right]^{{\text{T}}} } \right)_{,\zeta } } \right)\left\{ \sigma \right\}{\text{d}}\eta {\text{d}}\zeta } \\ \end{array} } \right)d\xi } \\ \end{array} \\ & = \mathop \int \limits_{0}^{1} \begin{array}{*{20}c} {\xi \left( {\begin{array}{*{20}c} {\mathop {\oint }\limits_{{\Gamma^{\xi } }} \left[ {w_{j} } \right]^{{\text{T}}} \left( {\left\{ {t^{\eta } } \right\}g^{\eta } {\text{d}}\zeta + \left\{ {t^{\zeta } } \right\}g^{\zeta } {\text{d}}\eta } \right)} \\ { - \mathop {\iint }\limits_{{S^{\xi } }} \left( {\begin{array}{*{20}c} {\left[ {w_{j} } \right]^{{\text{T}}} \left( {\left| {\left[ {\hat{J}} \right]} \right|\left[ {b^{2} } \right]^{{\text{T}}} } \right)_{,\eta } + \left[ {w_{j} } \right]_{,\eta }^{T} \left| {\left[ {\hat{J}} \right]} \right|\left[ {b^{2} } \right]^{{\text{T}}} } \\ { + \left[ {w_{j} } \right]^{{\text{T}}} \left( {\left| {\left[ {\hat{J}} \right]} \right|\left[ {b^{3} } \right]^{{\text{T}}} } \right)_{,\zeta } + \left[ {w_{j} } \right]_{,\zeta }^{T} \left| {\left[ {\hat{J}} \right]} \right|\left[ {b^{3} } \right]^{{\text{T}}} } \\ \end{array} } \right)\left\{ \sigma \right\}{\text{d}}\eta {\text{d}}\zeta } \\ \end{array} } \right)d\xi } \\ \end{array} \\ & = \mathop \int \limits_{0}^{1} \begin{array}{*{20}c} {\xi \left( {\begin{array}{*{20}c} {\mathop {\oint }\limits_{{\Gamma^{\xi } }} \left[ {w_{j} } \right]^{{\text{T}}} \left( {\left\{ {t^{\eta } } \right\}g^{\eta } {\text{d}}\zeta + \left\{ {t^{\zeta } } \right\}g^{\zeta } {\text{d}}\eta } \right)} \\ { - \mathop {\iint }\limits_{{S^{\xi } }} \left( {\begin{array}{*{20}c} {\left[ {w_{j} } \right]^{{\text{T}}} \left( {\left( {\left| {\left[ {\hat{J}} \right]} \right|\left[ {b^{2} } \right]^{{\text{T}}} } \right)_{,\eta } + \left( {\left| {\left[ {\hat{J}} \right]} \right|\left[ {b^{3} } \right]^{{\text{T}}} } \right)_{,\zeta } } \right)} \\ { + \left[ {w_{j} } \right]_{,\eta }^{T} \left| {\left[ {\hat{J}} \right]} \right|\left[ {b^{2} } \right]^{{\text{T}}} + \left[ {w_{j} } \right]_{,\zeta }^{T} \left| {\left[ {\hat{J}} \right]} \right|\left[ {b^{3} } \right]^{{\text{T}}} } \\ \end{array} } \right)\left\{ \sigma \right\}{\text{d}}\eta {\text{d}}\zeta } \\ \end{array} } \right)d\xi } \\ \end{array} , \\ \end{aligned}$$

where the surface traction boundary condition $\left\{ {t^{\eta } } \right\}$ and $\left\{ {t^{\zeta } } \right\}$ is introduced naturally.

Substituting Eq. (34) into Eq. (51) results in

52 $$\begin{aligned} {\text{II}} & = \mathop \int \limits_{0}^{1} \begin{array}{*{20}c} {\xi \left( {\begin{array}{*{20}c} {\mathop {\oint }\limits_{{\Gamma^{\xi } }} \left[ {w_{j} } \right]^{{\text{T}}} \left( {\left\{ {t^{\eta } } \right\}g^{\eta } {\text{d}}\zeta + \left\{ {t^{\zeta } } \right\}g^{\zeta } {\text{d}}\eta } \right)} \\ { - \mathop {\iint }\limits_{{S^{\xi } }} \left( {\begin{array}{*{20}c} {\left[ {w_{j} } \right]^{{\text{T}}} \left( {\left( {\left| {\left[ {\hat{J}} \right]} \right|\left[ {b^{2} } \right]^{{\text{T}}} } \right)_{,\eta } + \left( {\left| {\left[ {\hat{J}} \right]} \right|\left[ {b^{3} } \right]^{{\text{T}}} } \right)_{,\zeta } } \right)} \\ { + \left[ {w_{j} } \right]_{,\eta }^{T} \left| {\left[ {\hat{J}} \right]} \right|\left[ {b^{2} } \right]^{{\text{T}}} + \left[ {w_{j} } \right]_{,\zeta }^{T} \left| {\left[ {\hat{J}} \right]} \right|\left[ {b^{3} } \right]^{{\text{T}}} } \\ \end{array} } \right)\left\{ \sigma \right\}{\text{d}}\eta {\text{d}}\zeta } \\ \end{array} } \right)d\xi } \\ \end{array} \\ & = \mathop \int \limits_{0}^{1} \begin{array}{*{20}c} {\xi \left( {\begin{array}{*{20}c} {\mathop {\oint }\limits_{{\Gamma^{\xi } }} \left[ {w_{j} } \right]^{{\text{T}}} \left( {\left\{ {t^{\eta } } \right\}g^{\eta } {\text{d}}\zeta + \left\{ {t^{\zeta } } \right\}g^{\zeta } {\text{d}}\eta } \right)} \\ { - \mathop {\iint }\limits_{{S^{\xi } }} \left( {\begin{array}{*{20}c} {\left[ {w_{j} } \right]^{{\text{T}}} \left( { - 2\left| {\left[ {\hat{J}} \right]} \right|\left[ {b^{1} } \right]^{{\text{T}}} } \right) + \left[ {w_{j} } \right]_{,\eta }^{T} \left| {\left[ {\hat{J}} \right]} \right|\left[ {b^{2} } \right]^{{\text{T}}} } \\ { + \left[ {w_{j} } \right]_{,\zeta }^{T} \left| {\left[ {\hat{J}} \right]} \right|\left[ {b^{3} } \right]^{{\text{T}}} } \\ \end{array} } \right)\left\{ \sigma \right\}{\text{d}}\eta {\text{d}}\zeta } \\ \end{array} } \right)d\xi } \\ \end{array} \\ & = \mathop \int \limits_{0}^{1} \begin{array}{*{20}c} {\xi \left( {\begin{array}{*{20}c} {\mathop {\oint }\limits_{{\Gamma^{\xi } }} \left[ {w_{j} } \right]^{{\text{T}}} \left( {\left\{ {t^{\eta } } \right\}g^{\eta } {\text{d}}\zeta + \left\{ {t^{\zeta } } \right\}g^{\zeta } {\text{d}}\eta } \right)} \\ { - \mathop {\iint }\limits_{{S^{\xi } }} \left( {\begin{array}{*{20}c} { - 2\left[ {w_{j} } \right]^{{\text{T}}} \left[ {b^{1} } \right]^{{\text{T}}} + \left[ {w_{j} } \right]_{,\eta }^{T} \left[ {b^{2} } \right]^{{\text{T}}} } \\ { + \left[ {w_{j} } \right]_{,\zeta }^{T} \left[ {b^{3} } \right]^{{\text{T}}} } \\ \end{array} } \right)\left\{ \sigma \right\}\left| {\left[ {\hat{J}} \right]} \right|{\text{d}}\eta {\text{d}}\zeta } \\ \end{array} } \right)d\xi } \\ \end{array} . \\ \end{aligned}$$

Substituting Eq. (52) into Eq. (47), the equivalent integral weak form of Eq. (47) is given by

53 $$\begin{aligned} & \xi^{2} \mathop \int \limits_{ - 1}^{1} \mathop \int \limits_{ - 1}^{1} \left[ {w_{j} } \right]^{{\text{T}}} \left[ {b^{1} } \right]^{{\text{T}}} \left\{ \sigma \right\}_{,\xi } \left| {\left[ {\hat{J}} \right]} \right|d\eta d\zeta \\ & \quad + \xi \mathop {\oint }\limits_{{\Gamma^{\xi } }} \begin{array}{*{20}c} {\left[ {w_{j} } \right]^{{\text{T}}} \left( {\left\{ {t^{\eta } } \right\}g^{\eta } {\text{d}}\zeta + \left\{ {t^{\zeta } } \right\}g^{\zeta } {\text{d}}\eta } \right)} \\ \end{array} \\ & \quad - \xi \mathop \int \limits_{ - 1}^{1} \begin{array}{*{20}c} {\begin{array}{*{20}c} {\begin{array}{*{20}c} {\mathop \int \limits_{ - 1}^{1} \left( { - 2\left[ {w_{j} } \right]^{{\text{T}}} \left[ {b^{1} } \right]^{{\text{T}}} + \left[ {w_{j} } \right]_{,\eta }^{T} \left[ {b^{2} } \right]^{{\text{T}}} + \left[ {w_{j} } \right]_{,\zeta }^{T} \left[ {b^{3} } \right]^{{\text{T}}} } \right)\left\{ \sigma \right\}\left| {\left[ {\hat{J}} \right]} \right|d\eta d\zeta } \\ \end{array} } \\ \end{array} } \\ \end{array} \\ & \quad + \xi^{2} \mathop \int \limits_{ - 1}^{1} \mathop \int \limits_{ - 1}^{1} \left[ {w_{j} } \right]^{{\text{T}}} \left\{ p \right\}\left| {\left[ {\hat{J}} \right]} \right|d\eta d\zeta \\ & \quad - \xi^{2} \mathop \int \limits_{ - 1}^{1} \mathop \int \limits_{ - 1}^{1} \left[ {w_{j} } \right]^{{\text{T}}} \rho \left\{ {\ddot{u}} \right\}\left| {\left[ {\hat{J}} \right]} \right|d\eta d\zeta = \left\{ 0 \right\}, \\ \end{aligned}$$

where the surface traction boundary condition $\left\{ {t^{\eta } } \right\}$ and $\left\{ {t^{\zeta } } \right\}$ is introduced naturally, $\Gamma^{\xi }$ denotes the the boudnary of surafce $S^{\xi }$.

By applying the weighted residual method, the arbitrary weight function $\left[ {w_{j} } \right]$ in Eq. (53) is simplified to a particular weight function $\left\{ w \right\}$, the approximate form of Eq. (53) is given by

54 $$\begin{aligned} & \xi^{2} \mathop \int \limits_{ - 1}^{1} \mathop \int \limits_{ - 1}^{1} \left\{ w \right\}^{{\text{T}}} \left[ {b^{1} } \right]^{{\text{T}}} \left\{ \sigma \right\}_{,\xi } \left| {\left[ {\hat{J}} \right]} \right|d\eta d\zeta \\ & \quad + \xi \mathop {\oint }\limits_{{\Gamma^{\xi } }} \begin{array}{*{20}c} {\left\{ w \right\}^{{\text{T}}} \left( {\left\{ {t^{\eta } } \right\}g^{\eta } {\text{d}}\zeta + \left\{ {t^{\zeta } } \right\}g^{\zeta } {\text{d}}\eta } \right)} \\ \end{array} \\ & \quad - \xi \mathop \int \limits_{ - 1}^{1} \begin{array}{*{20}c} {\begin{array}{*{20}c} {\begin{array}{*{20}c} {\mathop \int \limits_{ - 1}^{1} \left( { - 2\left\{ w \right\}^{{\text{T}}} \left[ {b^{1} } \right]^{{\text{T}}} + \left\{ w \right\}_{,\eta }^{T} \left[ {b^{2} } \right]^{{\text{T}}} + \left\{ w \right\}_{,\zeta }^{T} \left[ {b^{3} } \right]^{{\text{T}}} } \right)\left\{ \sigma \right\}\left| {\left[ {\hat{J}} \right]} \right|d\eta d\zeta } \\ \end{array} } \\ \end{array} } \\ \end{array} \\ & \quad + \xi^{2} \mathop \int \limits_{ - 1}^{1} \mathop \int \limits_{ - 1}^{1} \left\{ w \right\}^{{\text{T}}} \left\{ p \right\}\left| {\left[ {\hat{J}} \right]} \right|d\eta d\zeta \\ & \quad - \xi^{2} \mathop \int \limits_{ - 1}^{1} \mathop \int \limits_{ - 1}^{1} \left\{ w \right\}^{{\text{T}}} \rho \left\{ {\ddot{u}} \right\}\left| {\left[ {\hat{J}} \right]} \right|d\eta d\zeta = \left\{ 0 \right\}, \\ \end{aligned}$$

where $\left\{ w \right\}^{{\text{T}}}$ denotes the weight functions in the W-element, $\left\{ \cdot \right\}^{{\text{T}}}$ denotes a row vector.

Considering the Galerkin weighted residual method, the interpolation of $\left\{ w \right\}^{{\text{T}}}$ is the same as $\left\{ {u\left( {\xi , \eta , \zeta } \right)} \right\}$ in Eq. (43) as

55 $$\left\{ {w\left( {\xi , \eta , \zeta } \right)} \right\} = \left[ {N\left( {\eta , \zeta } \right)} \right]\left\{ {w^{e} \left( \xi \right)} \right\}.$$

Substituting Eq. (43), Eqs. (29) and (55) into Eq. (54), the SBFEM governing equation in the time domain for 3D elastic continuum media is given by^53^

56 $$\begin{aligned} & \left[ {E^{0} } \right]\xi^{2} \left\{ u \right\}_{,\xi \xi } + \left( {2\left[ {E^{0} } \right] - \left[ {E^{1} } \right] + \left[ {E^{1} } \right]^{{\text{T}}} } \right)\xi \left\{ u \right\}_{,\xi } + \left( {\left[ {E^{1} } \right]^{{\text{T}}} - \left[ {E^{2} } \right]} \right)\left\{ u \right\} \\ & \quad - \left[ {M^{0} } \right]\xi^{2} \left\{ {\ddot{u}^{e} \left( \xi \right)} \right\} + \xi \left\{ T \right\} + \xi^{2} \left\{ P \right\} = \left\{ 0 \right\}, \\ \end{aligned}$$

where the constant coefficient matrix is given by

57 $$\begin{aligned} \left[ {E^{0} } \right] & = \mathop \int \limits_{ - 1}^{1} \mathop \int \limits_{ - 1}^{1} \left[ {B^{1} } \right]^{{\text{T}}} \left[ D \right]\left[ {B^{1} } \right]\left| {\left[ {\hat{J}} \right]} \right|d\eta d\zeta , \\ \left[ {E^{1} } \right] & = \mathop \int \limits_{ - 1}^{1} \mathop \int \limits_{ - 1}^{1} \left[ {B^{2} } \right]^{{\text{T}}} \left[ D \right]\left[ {B^{1} } \right]\left| {\left[ {\hat{J}} \right]} \right|d\eta d\zeta , \\ \left[ {E^{2} } \right] & = \mathop \int \limits_{ - 1}^{1} \mathop \int \limits_{ - 1}^{1} \left[ {B^{2} } \right]^{{\text{T}}} \left[ D \right]\left[ {B^{2} } \right]\left| {\left[ {\hat{J}} \right]} \right|d\eta d\zeta , \\ \left[ {M^{0} } \right] & = \mathop \int \limits_{ - 1}^{1} \mathop \int \limits_{ - 1}^{1} \left[ N \right]^{{\text{T}}} \rho \left[ N \right]\left| {\left[ {\hat{J}} \right]} \right|d\eta d\zeta . \\ \end{aligned}$$

The governing equation of W-elements sharing the same scaling center can be assembled by the degree of freedom contributed by adjacent W-elements, which results in the governing equation of the S-element like Eq. (56) but with the assembled $\left\{ u \right\}$, $\left[ {E^{0} } \right]$, $\left[ {E^{1} } \right]$, $\left[ {E^{2} } \right]$, and $\left[ {M^{0} } \right]$.

### Solution of the SBFEM governing equation

The SBFEM governing equation (Eq. (56)) is a system of second order Ordinary Differential Equations (ODEs) called the Euler-Cauchy equations. The solution of this system is given in a closed form. The first step is to introduce a dual variable to convert the system of second order ODEs to a system of one order with variables doubled in size. Based on the principle of virtual work, the internal node force $\left\{ {q\left( \xi \right)} \right\}$ along the ray starting from the scaling center to the nodes on the boundary resulting from the surface traction $\left\{ {t^{\xi } } \right\}$ on the coordinate surface of $S^{\xi }$ is given by

58 $$\left\{ {w\left( \xi \right)} \right\}^{{\text{T}}} \left\{ {q\left( \xi \right)} \right\} = \mathop \int \limits_{{S^{\xi } }} \left\{ {w\left( {\xi , \eta , \zeta } \right)} \right\}^{{\text{T}}} \left\{ {t^{\xi } \left( {\xi , \eta , \zeta } \right)} \right\}dS^{\xi } ,$$

59 $$\left\{ {q\left( \xi \right)} \right\} = \left[ {E^{0} } \right]\xi^{2} \left\{ {u\left( \xi \right)} \right\}_{,\xi } + \left[ {E^{1} } \right]^{{\text{T}}} \xi \left\{ {u\left( \xi \right)} \right\}.$$

If the body forces vanish, Eq. (56) can be reformulated as

60 $$\xi \left\{ {X\left( \xi \right)} \right\}_{,\xi } = \left[ Z \right]\left\{ {X\left( \xi \right)} \right\}.$$

where

61 $$\left\{ {X\left( \xi \right)} \right\} = \left\{ {\begin{array}{*{20}c} {\xi^{0.5} \left\{ {u\left( \xi \right)} \right\}} \\ {\xi^{ - 0.5} \left\{ {q\left( \xi \right)} \right\}} \\ \end{array} } \right\},$$

and

62 $$\left[ Z \right] = \left[ {\begin{array}{*{20}c} {0.5\left[ I \right] - \left[ {E^{0} } \right]^{ - 1} \left[ {E^{1} } \right]^{{\text{T}}} } & {\left[ {E^{0} } \right]^{ - 1} } \\ {\left[ {E^{2} } \right] - \left[ {E^{1} } \right]\left[ {E^{0} } \right]^{ - 1} \left[ {E^{1} } \right]^{{\text{T}}} } & {\left[ {E^{1} } \right]\left[ {E^{0} } \right]^{ - 1} - 0.5\left[ I \right]} \\ \end{array} } \right].$$

The eigenvalues and eigenvectors of the matrix $\left[ Z \right]$ can be obtained by

63 $$\left[ Z \right]\left[ \Phi \right] = \left[ \Phi \right]\left[ \lambda \right],{ }\left[ \lambda \right] = {\text{diag}}\left( {\lambda_{1} , \lambda_{2} , \ldots , \lambda_{2n} } \right),$$

where $$\left[ \Phi \right] = \left[ {\begin{array}{*{20}c} {\left\{ {\phi_{1} } \right\}} & {\left\{ {\phi_{1} } \right\}} & \ldots & {\left\{ {\phi_{2n} } \right\}} \\ \end{array} } \right]$$ denotes a matrix consisted of the eigenvectors $$\begin{array}{*{20}c} {\left\{ {\phi_{1} } \right\},} & {\left\{ {\phi_{1} } \right\},} & { \ldots ,} & {\left\{ {\phi_{2n} } \right\}} \\ \end{array}$$, $\lambda_{1} , \lambda_{2} , \ldots , \lambda_{2n}$ denotes the eigenvalues, ${\text{diag}}\left( \cdot \right)$ denotes a diagonal matrix. It is worth to notice that the eigenvalues in $\left[ \lambda \right]$ should be in descending order, so the eigenvectors in $\left[ \Phi \right]$. The eigenvectors $$\begin{array}{*{20}c} {\left\{ {\phi_{1} } \right\},} & {\left\{ {\phi_{1} } \right\},} & { \ldots ,} & {\left\{ {\phi_{2n} } \right\}} \\ \end{array}$$ and the eigenvalues $\lambda_{1} , \lambda_{2} , \ldots ,\lambda_{2n}$ can be partitioned into two blocks as

64 $$\left[ \Phi \right] = \left[ {\begin{array}{*{20}c} {\left[ {\Phi_{b}^{u} } \right]} \\ {\left[ {\Phi_{b}^{q} } \right]} \\ \end{array} } \right],$$

65 $$\left[ \lambda \right] = \left[ {\begin{array}{*{20}c} {\left[ {\lambda^{u} } \right]} & {\left[ {0^{u} } \right]} \\ {\left[ {0^{q} } \right]} & {\left[ {\lambda^{q} } \right]} \\ \end{array} } \right],$$

where the subscript $u$ denotes the block related to $\left\{ {u\left( \xi \right)} \right\}$, the subscript $q$ denotes the block related to $\left\{ {q\left( \xi \right)} \right\}$.

The solution of Eq. (60) considering boundary conditions is given by

66 $$\left\{ {u\left( \xi \right)} \right\} = \xi^{ - 0.5} \left[ {\Phi_{b}^{u} } \right]\xi^{{\lambda_{b} }} \left\{ c \right\},$$

67 $$\left\{ {q\left( \xi \right)} \right\} = \xi^{0.5} \left[ {\Phi_{b}^{q} } \right]\xi^{{\lambda_{b} }} \left\{ c \right\},$$

where $$\left\{ c \right\} = \left[ {\begin{array}{*{20}c} {c_{1} } & {c_{2} } & \cdots & {c_{n} } \\ \end{array} } \right]^{{\text{T}}}$$ denotes the arbitrary integration constant, $\xi^{{\lambda_{b} }}$ denotes a diagonal matrix expressed as

68 $$\xi^{{\lambda_{b} }} = \left[ {\begin{array}{*{20}c} {\xi^{{\lambda_{1} }} } & 0 & \cdots & 0 \\ 0 & {\xi^{{\lambda_{2} }} } & \ddots & \vdots \\ \vdots & \ddots & \ddots & 0 \\ 0 & \cdots & 0 & {\xi^{{\lambda_{n} }} } \\ \end{array} } \right],$$

For the SBFEM polyhedron element, the coefficient matrix representing the stiffness and mass matrix of the S-element need to be derived. Substituting Eq. (67) into Eq. (66) to eliminate $\left\{ c \right\}$, the stiffness matrix $\left[ K \right]$ is given by^51^

69 $$\left[ K \right] = \left[ {\Phi_{b}^{q} } \right]\left[ {\Phi_{b}^{u} } \right]^{ - 1} ,$$

where $\left[ \cdot \right]^{ - 1}$ denotes the inverse of the matrix.

Based on the virtual work statement, the mass matrix $\left[ M \right]$ of the S-element can be expressed as^51^

70 $$\left[ M \right] = \left[ {\Phi_{b}^{u} } \right]^{{ - {\text{T}}}} \left[ m \right]\left[ {\Phi_{b}^{u} } \right]^{ - 1} ,$$

where $\left[ \cdot \right]^{{ - {\text{T}}}}$ denotes the inverse of the transpose of the matrix, the value $m_{ij}$ in the row $i$ and column $j$ of $\left[ m \right]$ is defined as

71 $$\left[ m \right] = m_{ij} = \frac{{m_{ij}^{0} }}{{\lambda_{i}^{b} + \lambda_{j}^{b} + 2}},$$

where the $m_{ij}^{0}$ is the value in the row $i$ and column $j$ of $\left[ {m^{0} } \right]$ defined as

72 $$m_{ij}^{0} = \left[ {m^{0} } \right] = \left[ {\Phi_{b}^{u} } \right]^{{ - {\text{T}}}} \left[ {M^{0} } \right]\left[ {\Phi_{b}^{u} } \right]^{ - 1} .$$

## Topology and data structure of an arbitrary polyhedron element

The polyhedron geometry is a 3D shape with straight edges, sharp corners and vertices, and any number greater than 2 of plane polygonal faces with any number greater than 2 of straight edges and vertices. The tetrahedron, pentahedron, hexahedron and heptahedron that widely used in FEM are just the special cases of the convex polyhedron. Vastly different from the FEM element geometry, the first step to construct a polyhedron element is to describe the uncertainty of the topology of varied kinds of polyhedron elements in a model with a uniform data structure that can be recorded in the intermediate file for compatible purpose.

### Essential information to describe the topology of an arbitrary polyhedron

**Figure 6:**
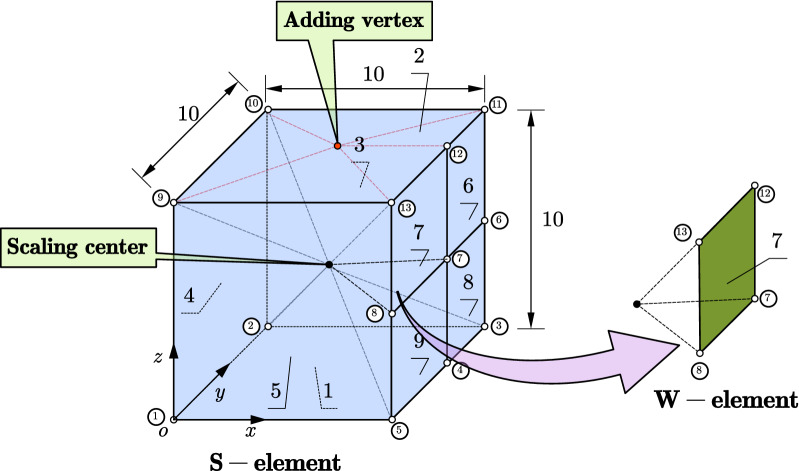
Topology of a polyhedron element and the treatment of the polygon facet.

For example, Fig. 6 shows a polyhedron element that may be encountered in an octree mesh, which has nine facets, twenty edges and thirteen vertices. The coordinates of the vertices are the basic information as shown in Table 1. In addition, the connectivity of the vertices of each facet needs to be defined as shown in Table 2. These two sets of information are sufficient and necessary for the description of the topology of any polyhedron. It is worth to notice that the order of the vertices and facets of a polyhedron is insignificant and when there are multiple polyhedra, there is no need to record the correspondence between the local and global vertex ID like FEM.

**Table 1:** Vertex information of the polyhedron element.

Vertex ID	X coordinates	Y coordinates	Z coordinates
1	0.0	0.0	0.0
2	0.0	10.0	0.0
3	10.0	10.0	0.0
4	10	5.0	0.0
5	10.0	0.0	0.0
6	10.0	10.0	5.0
7	10.0	5.0	5.0
8	10.0	0.0	5.0
9	0.0	0.0	10.0
10	0.0	10.0	10.0
11	10.0	10.0	10.0
12	10.0	5.0	0.0
13	10.0	0.0	10.0

**Table 2:** Facet information of the polyhedron.

Facet ID	Vertex connectivity
1	1, 2, 3, 4, 5
2	9, 13, 12, 11, 10
3	2, 10, 11, 6, 3
4	1, 9, 10, 2
5	1, 5, 8, 13, 9
6	7, 6, 11, 12
7	8, 7, 12, 13
8	4, 3, 6, 7
9	5, 4, 7, 8

### SBFEM discretization of the polyhedron

The geometry of the S-element can be adapted for any convex polyhedron with only triangle or quadrilateral facets which is a special case of the polyhedron defined in “Essential information to describe the topology of an arbitrary polyhedron” section. Nevertheless, the polygonal facet of a polyhedron can always be converted into multiple triangle or quadrilateral facets by adding a vertex on this facet (see Fig. 6). After this treatment of the polyhedron facets, a point inside the polyhedron that can see all the points on the facets is chosen as the scaling center and the vertices are defined as nodes of this polyhedron domain. The scaling center and the nodes of each facet constitute a W-element. All these W-elements are assembled into an S-element representing this polyhedron domain. It is obvious that the number of W-elements is equal to the number of facets of the polyhedron and the number of the nodes of the S-element is equal to the number of vertices of the polyhedron. The data structure to describe any 3D SBFEM polyhedron element is summarized in Table 3.

**Table 3:** Data structure of the SBFEM polyhedron element.

Element ID	*k*	
Coordinate of scaling center	*x*_0_, *y*_0_, *z*_0_	
Number of facets	*m*	
Number of nodes	*n*	

Local node ID	Global node ID	Node coordinates
1	*N*_1_	*x*_1_, *y*_1_, *z*_1_
2	*N*_2_	*x*_2_, *y*_2_, *z*_2_
**…**	…	…
*n*	*N*_*n*_	*x*_*n*_, *y*_*n*_, *z*_*n*_

Facets	Number of nodes	Node conectivity^a^
1	*J*_1_	*N*_11_, *N*_12_, …,$N_{{1J_{1} }}$
2	*J*_2_	*N*_11_, *N*_12_, …,$N_{{1J_{2} }}$
…	…	…
*m*	*J*_*m*_	*N*_*m*1_, *N*_*m*2_, …,$N_{{mJ_{m} }}$

Density	$\rho_{k}$	
Young’s modulus	*E*_*k*_	
Poisson’s ratio	*v*_*k*_	

^a^The node sequence should be in a certain order pointing out of the region according to the right-hand rule.

### Generation of the SBFEM polyhedron mesh

**Figure 7:**
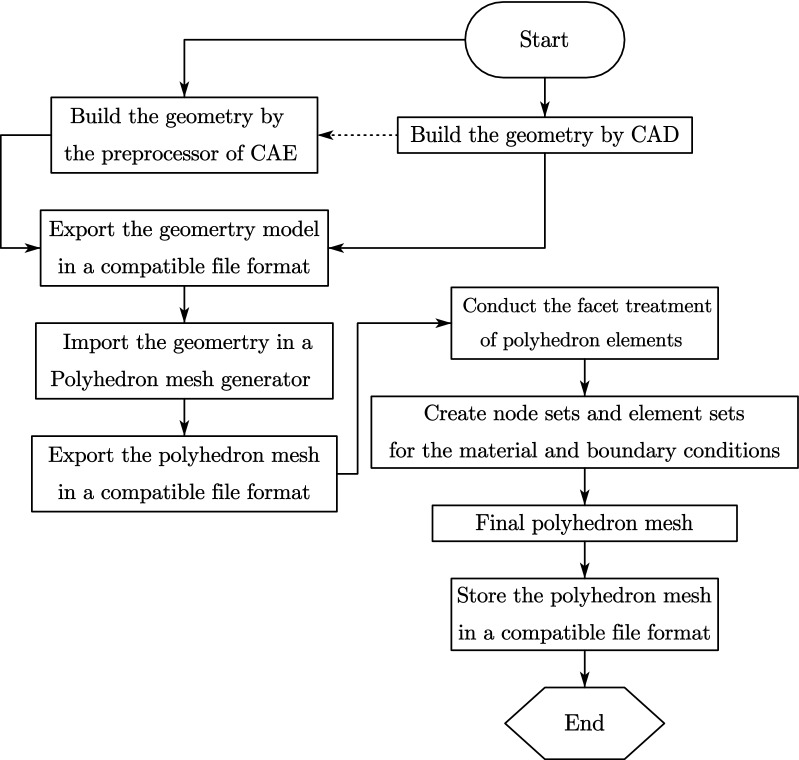
General procedure of SBFEM polyhedron mesh generation by out-of-box tools.

There are roughly three kinds of tools to obtain a polyhedron mesh: the self-developed program, the open-source or commercial mesh generator. Liu et al. brought up an automatic octree mesh generation algorithm from an STL geometry file in MATLAB for three-dimensional domain^38^. Besides, at present OPENFOAM, FLUENT and STAR-CCM + provide robust polyhedron mesh generators, the latter two of which are the most convenient. The general procedure to generate a polyhedron mesh from geometry with an out-of-box tool is illustrated in Fig. 7. Because the common FEM software for solid media does not support the polyhedron element, the preprocessor built in them cannot generate polyhedron meshes. Furthermore, the raw polyhedron mesh needs some extra processing steps such as classification, grouping, adding vertices before applied. The flow of geometry and mesh information between various software through files in compatible formats is inevitable. The Initial Graphics Exchange Specification (IGES) for the geometry information and Visualization Toolkit (VTK) for the mesh information are recommended. It should be noted that no matter which polyhedron mesh generator above is chosen or developed, it is required to store the polyhedron mesh in the intermediate file that will be imported into the UEL after the polyhedron mesh is obtained.

## Implementation in ABAQUS

The SBFEM polyhedron element is introduced in ABAQUS/Standard as a user element. The user element is implemented on the element iteration level of the solver in the form of a FORTRAN subroutine named UEL. During each increment of the computation, ABAQUS will invoke the UEL when calculating the coefficient matrix of each user element on and on until the end of the analysis. Assembly of the global coefficient matrix, nonlinear iteration, result output, imposition of contact, constrain, boundary and load conditions are performed by ABAQUS automatically.

### Basics of UEL in ABAQUS

UEL is essentially a function to compute specific variables with given variables. The subroutine name, declaration of the input and output variables are fixed (see “Appendix 1”^54^). During the calculation of each element, the UEL will be called importing information of analysis type, time increment, element type, right-hand-side vectors, etc. to export the mass matrix, stiffness matrix, equivalent nodes force vector, solution-dependent state variables, etc.

There are some conventions of user subroutines in ABAQUS that should be emphasized. All user subroutines and the subroutines created by users must be stored in a single source code file or its compiled object file. By default, the user subroutine can call another subroutine that is built in FORTRAN compiler, created by users or provided by ABAQUS named utility routines. This restriction suggests that some complicated calculations in a user subroutine have to be implemented by source codes provided by users. And if there were multiple user elements, these user elements should be implemented by the same subroutine of UEL with conditional branch statements on element types. Although user subroutines cannot call each other, the data can be transferred through the user-defined COMMON block variables between them.

### Associated INP and intermediate file

A convenient characteristic of ABAQUS is that the entire information of a FEM model is stored in an INP file, which is human-readable. This INP file is the only input required by the solver of ABAQUS. The user elements must be declared in the INP file before the calculation (see “Appendix 2”^54^). The declaration of user elements provide the information of the element type, element set name, number of nodes, node connectivity, node degrees of freedom and element properties such as material parameters, etc. According to the format of the user element declaration in an INP file, the user elements need to be classified into different types based on at least the number of nodes and the material properties and the node connectivity of polygonal facets of a polyhedron cannot be represented.

Due to the limitation of the INP file mentioned before, an intermediate file storing the topology information and material properties of arbitrary polyhedron elements is necessary. This file should be imported once the calculation is started and when iterating through the elements, the corresponding topology information of current user element should be introduced into UEL. With this intermediate file, any polyhedron mesh generator mentioned in “Generation of the SBFEM polyhedron mesh” section can be used and any mesh with arbitrary convex polyhedron shape can be calculated.

### Algorithm of UEL for SBFEM polyhedron element

**Figure 8:**
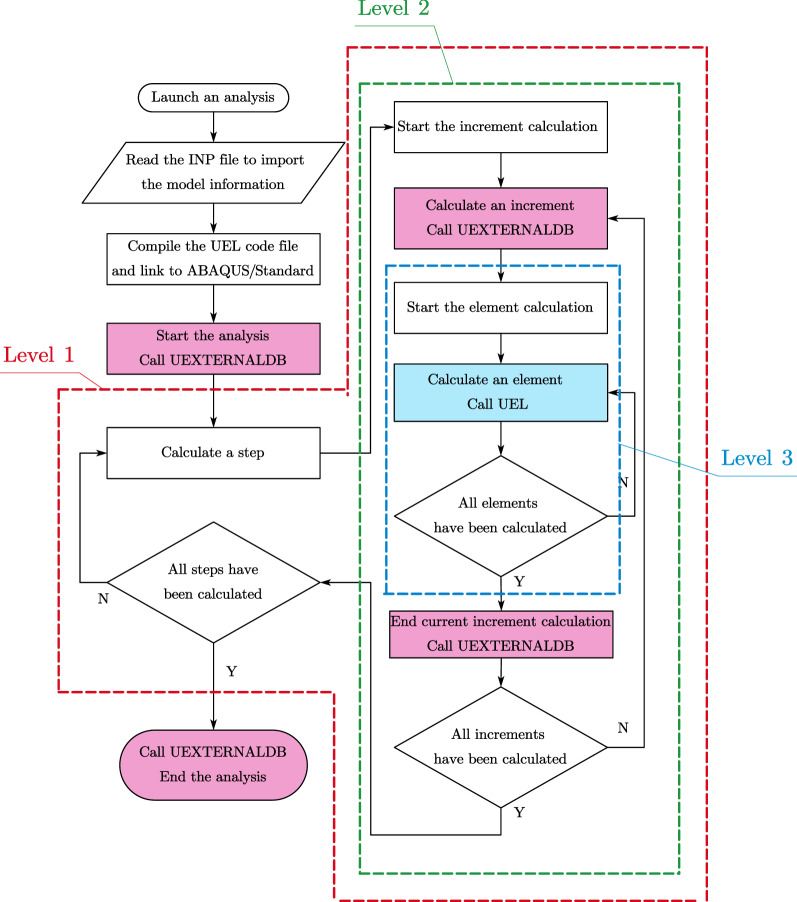
Hierarchy of the calculation process of ABAQUS/Standard.

Although UEL has provided a specific interface, it is necessary to understand the calculation process of ABAQUS/Standard to get a clear picture of what information is passed in UEL and what information UEL should return under different circumstances. As shown in Fig. 8, calculation process of ABAQUS/Standard has a hierarchy of three levels. Level 1 denotes the calculation procedure of steps in an analysis. Level 2 denotes the calculation procedure of increments of a step. Level 3 denotes the actual calculation of the polyhedron element matrix and vector in an increment by calling UEL for each element (see the green block in Fig. 8). The utility routine of UEXTERNALDB plays an important role in Level 1 and 2 (see the red blocks in Fig. 8), providing adequate interventions into the different phases of the calculation process, which give us more freedom to realize more complex functions. To coincide with UEL, the intermediate file storing the topology and material information of user polyhedron elements is imported by subroutine UEXTERNALDB at the beginning of the analysis, which is then transferred into UEL by COMMON block variables when the calculation of the polyhedron element starts.

**Figure 9:**
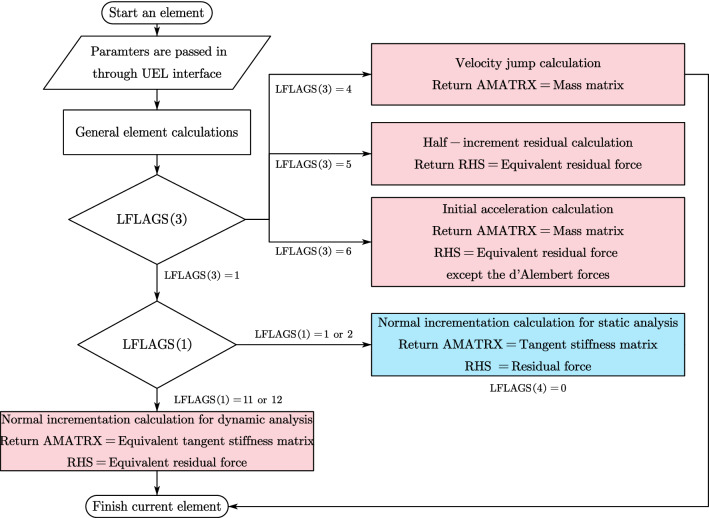
Basic branching structure of UEL for general static and direct-integration dynamic analysis.

The behavior of UEL is controlled by the basic branching structure (see Fig. 9) judged by the variable of LFLAGS. Considering only the general static and direct-integration dynamic analysis, there are five different cases (see colored blocks in Fig. 9) requiring returned values respectively according to the Newton–Raphson Method (NRM) and HHT direct time integration^55^.

The polyhedron element computation algorithm of UEL based on SBFEM is summarized in “Appendix 3”. The assembly of the coefficient matrix of W-elements in STEP 2.3 and stiffness and mass matrix calculation that include Eigen-decomposition are the major differences from FEM. Subroutines from LAPACK^56^ is appended to UEL in the code file to perform STEP 3. It is obvious that this UEL is compatible with the traditional FEM element topology built in ABAQUS, considering the tetrahedron, pentahedron and hexahedron as special cases of the polyhedron.

## Numerical application

There are two examples for the test and application of the implementation of arbitrary polyhedral elements developed in “Implementation in ABAQUS” section. A cantilever subjected to a harmonic excitation with the traditional hexahedron element and the polyhedron element are compared to confirm the accuracy of the developed UEL. Then the polyhedron element based on SBFEM is applied in the soil-structure dynamic interaction analysis of a nuclear power plant, the applicability and performance of this implementation is tested.

### Cantilever subjected to a harmonic excitation

**Figure 10:**
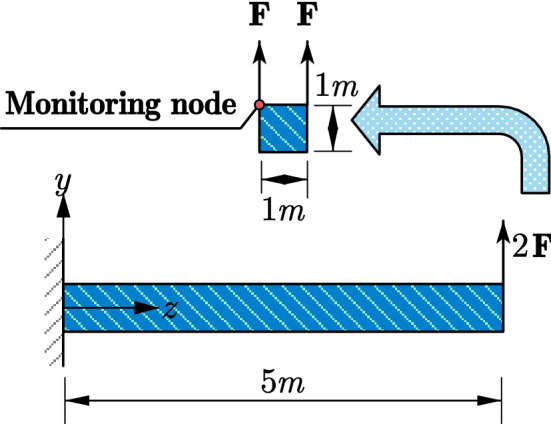
Geometry, load and boundary conditions of the cantilever.

A 5 m long horizontal beam subjected to concentrated forces on the vertices at one end and fixed at the other end is considered (see Fig. 10). The homogenous elastic material properties of Young’s modulus, density and Poisson’s ratio are 20 GPa, 2000 kg/m^3^, and 0.25 respectively. The harmonic concentrated forces in Y direction on the two vertices follow Eq. (73) defined as

73 $$F = A\sin \left( {\frac{2\pi }{T}t} \right),$$

where $T$ denoting the period is assuemd to be 0.05 s, $A$ denoting the amplitude is assumed to be 1000 N. $F$ denotes the concentrated force. The HHT direct time integration calculation is terminated at 0.25 s with a fixed time step of 0.001 s.

#### Numerical model

**Figure 11:**
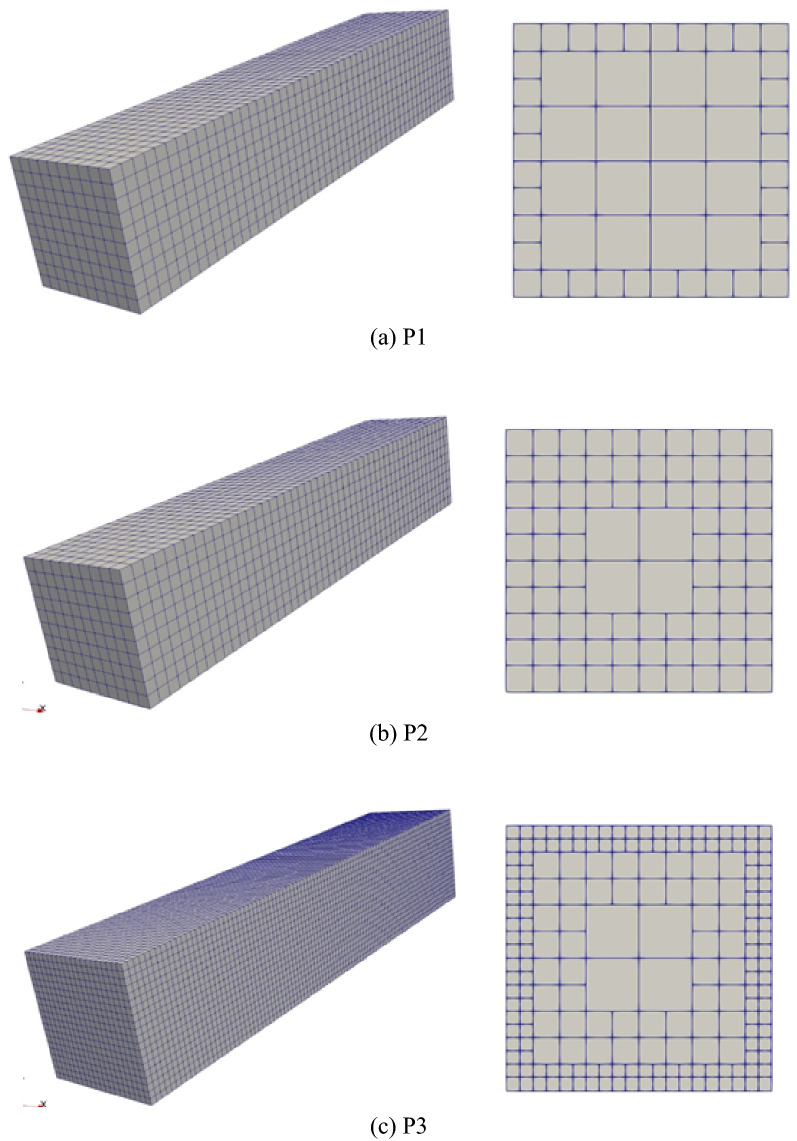
Polyhedron meshes of the cantilever. (**a**) P1, (**b**) P2, (**c**) P3.

**Figure 12:**
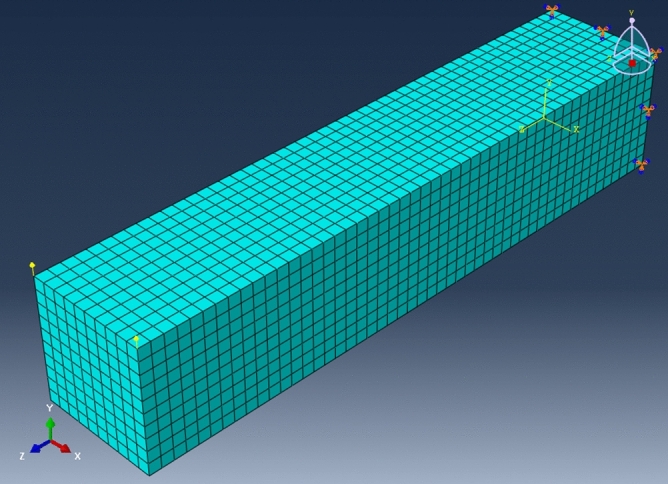
Hexahedron mesh of the cantilever (taken model H6 as an example).

From the geometry, two sets of meshes with varied mesh density are generated. The first set of meshes is made of structured hexahedron elements, namely C3D8 element (3D linear solid element with 8 nodes) built and verified in ABAQUS/Standard and the other is made of unstructured polyhedron elements implemented by UEL based on SBFEM. The details of the meshes are summarized in Table 4, where label P1 to P4 denote the polyhedron meshes with different mesh density (see Fig. 11) that can only be calculated by UEL developed in “Implementation in ABAQUS” section and H1 to H7 denote the hexahedron meshes (see Fig. 12) that can be calculated by C3D8 (built-in element) or the UEL (labeled as H2_UEL and H6_UEL). Model analysis for the first 50 modes of vibration and implicit dynamic analysis are performed.

**Table 4:** Mesh information of the cantilever.

Model name	Element number	Node number	Mesh type	Element type
P1	2312	4663	Polyhedron	UEL
P2	4384	5977	Polyhedron	UEL
P3	17,880	28,103	Polyhedron	UEL
H1	5	24	Hexahedron	C3D8
H2	40	99	Hexahedron	C3D8
H3	117	224	Hexahedron	C3D8
H4	153	288	Hexahedron	C3D8
H5	625	936	Hexahedron	C3D8
H6	5000	6171	Hexahedron	C3D8
H7	625,000	652,851	Hexahedron	C3D8
H2_UEL	40	99	Polyhedron	UEL
H6_UEL	5000	6171	Polyhedron	UEL

#### Results and discussion

**Figure 13:**
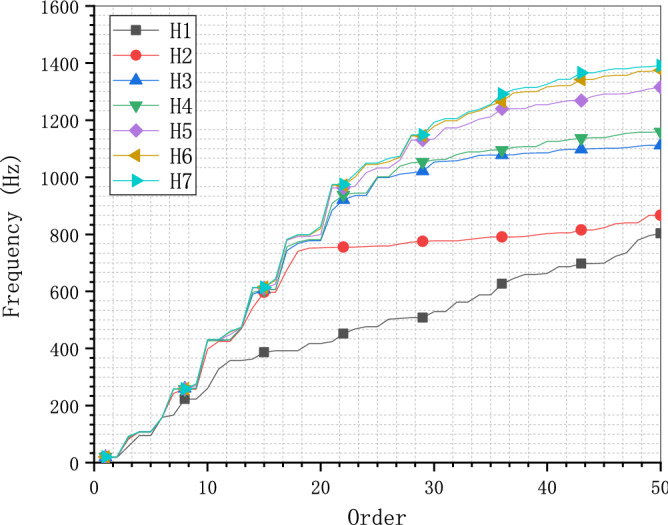
First 50 natural frequencies of the C3D8 meshes of the cantilever.

Model analysis checking the stiffness and mass matrix with specific boundary conditions is the most convenient approach to verify the user element for dynamic analysis, excluding the influence of load conditions and nonlinearity. The results of the first 50 natural frequencies and the displacement response of the dangling end (see Fig. 10) are compared to check the accuracy and efficiency of the UEL for the polyhedron element. As the increasing of the number of elements, the results of model analysis will converge to the true value of this problem. The first 50 natural frequencies of model H1 to H7 are listed in Fig. 13, from which the convergence values of the first 50 natural frequencies of the cantilever are obtained covering more than 95% effective mass in the main direction (97.1% in X and Y direction, 95.1% in Z direction).

**Figure 14:**
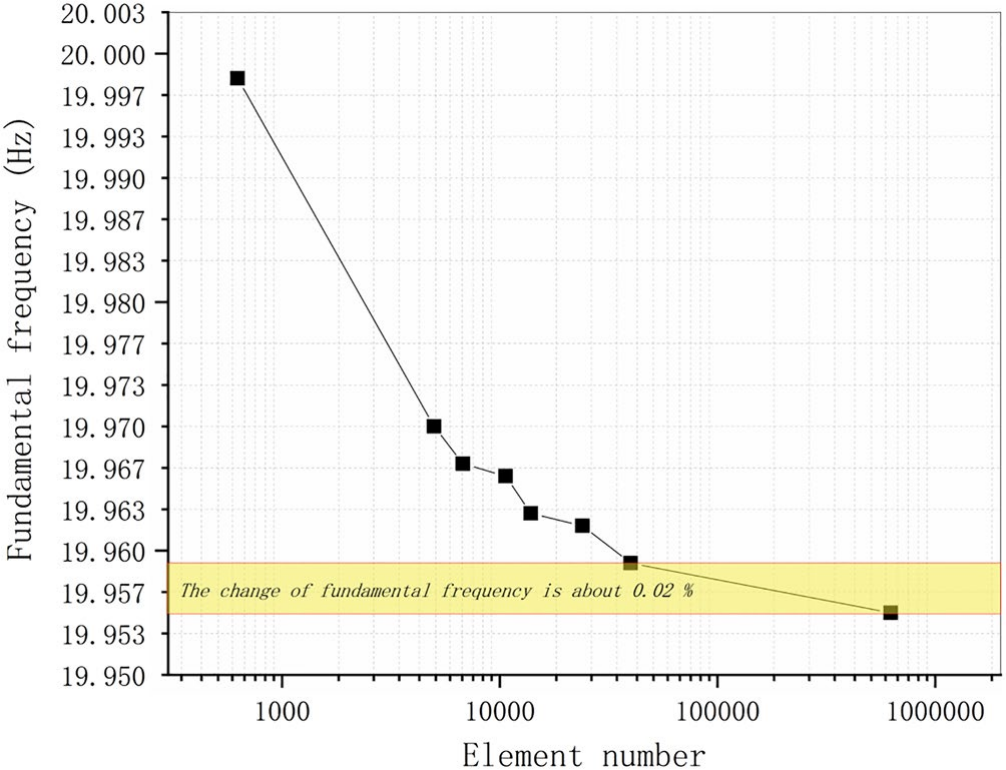
Fundamental frequencies of the C3D8 meshes of the cantilever.

Figure 13 shows that the difference between the frequencies of models with varied mesh densities is no more than 9% for the first vibration mode and this difference is enlarged to less than 19% for the first 9 vibration modes, which tends to keep on increasing with the order. Even though when the mesh is coarse, the convergence speed is fast with the increasing of the element, the convergence speed is much slower near the convergence values leading to rather poor computation efficiency (see Fig. 14). For example, the highest frequency of model H6 and H7 are 1374.8 Hz and 1392 Hz, the computation times of which are 5 s and 1109 s. More than 220 times the computation time of model H6 brings less than 1.2% improvement of the results of model H7. This is a typical illustration of the need to pay great attention to the balance between mesh density and calculation efficiency. Since it is reasonable to assume that the results of model H7 are close enough to the convergence values, the true fundamental frequency of this cantilever is assumed to be 19.955 Hz.

**Figure 15:**
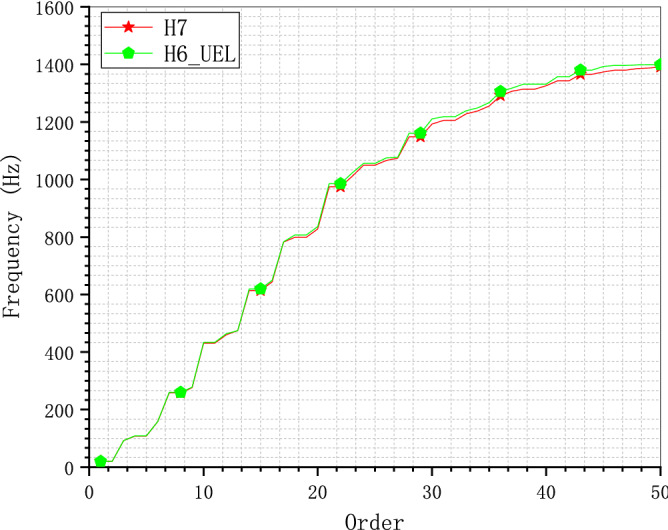
Frequency comparison of the C3D8 and UEL for mesh model H6.

**Figure 16:**
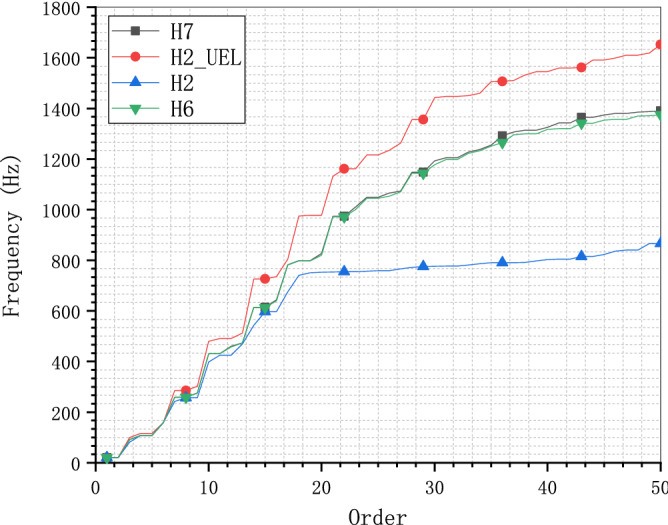
Frequency comparison of the C3D8 and UEL for mesh model H2.

To exclude the influence of the meshes, the same hexahedron meshes H2 and H6 are calculated by C3D8 and UEL respectively. Figure 15 shows the frequency comparison of the cantilever model H6_UEL calculated by UEL and H7 by C3D8. The consistency of the results of model H7 and H6_UEL confirms the accuracy of the UEL for the SBFEM-based polyhedron element with error less than 0.6% over the entire calculated frequency range. Figure 16 shows the frequency comparison of the cantilever mesh model H2 calculated by C3D8 and UEL. Compared to model H2 calculated by C3D8, model H2_UEL can get much better results that is almost equal to model H6 calculated by C3D8. In other words, polyhedron element based on SBFEM can get the same level of accuracy with less than 1% of the elements based on FEM, the most immediate advantage of which is the reduction in storage space for large-scale problems.

**Figure 17:**
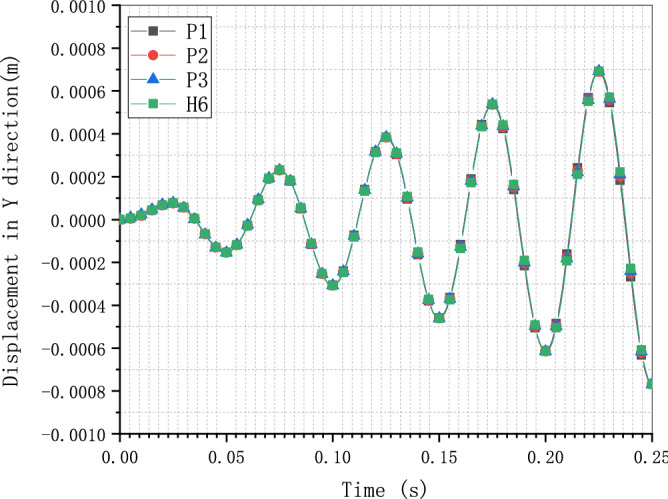
Displacement response in Y direction of models calculated by C3D8 and UEL.

The displacement responses in Y direction from the implicit dynamic analysis of model P1, P2, P3, and H6 are demonstrated in Fig. 17. Except the displacement values that are close to zero, the error between the model calculated by the C3D8 element and the user-defined polyhedron element based on SBFEM is less than 0.5%. The results of model analysis and direct dynamic analysis of the cantilever have proven the accuracy of the UEL implementation of the SBFEM-based polyhedron element and the efficiency of the SBFEM for the solid medium.

### Soil-structure dynamic interaction analysis of a nuclear power plant

**Figure 18:**
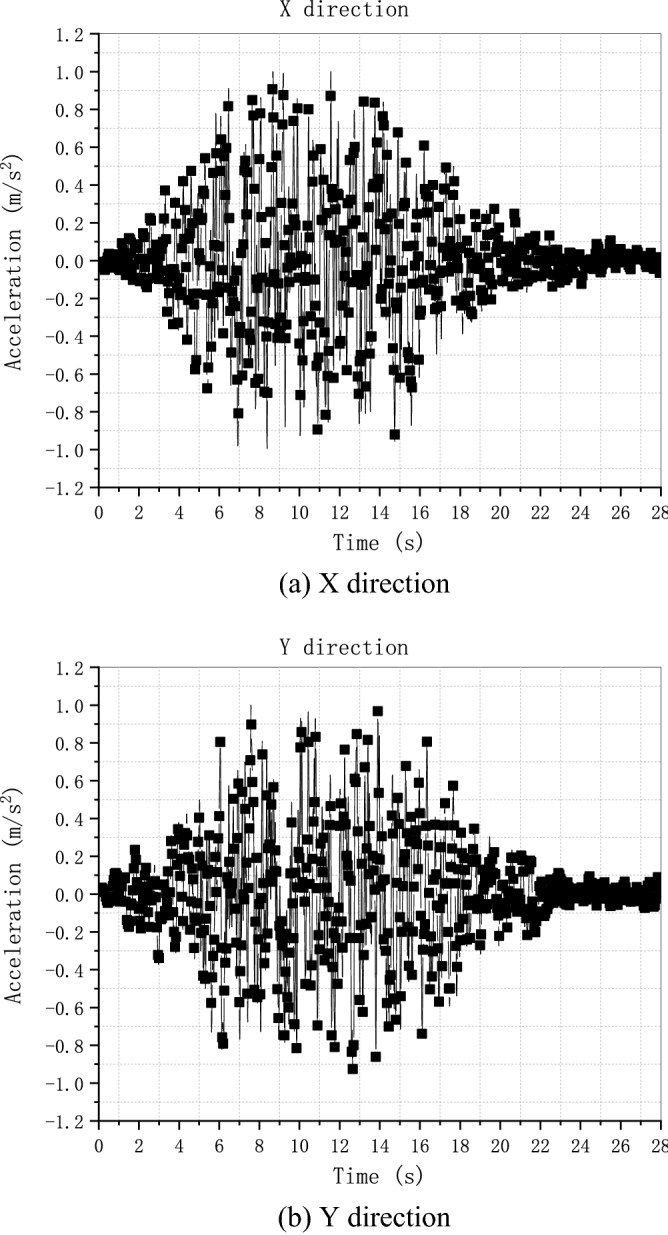
Artificial input earthquake in X and Y direction. (**a**) X direction, (**b**) Y direction.

**Figure 19:**
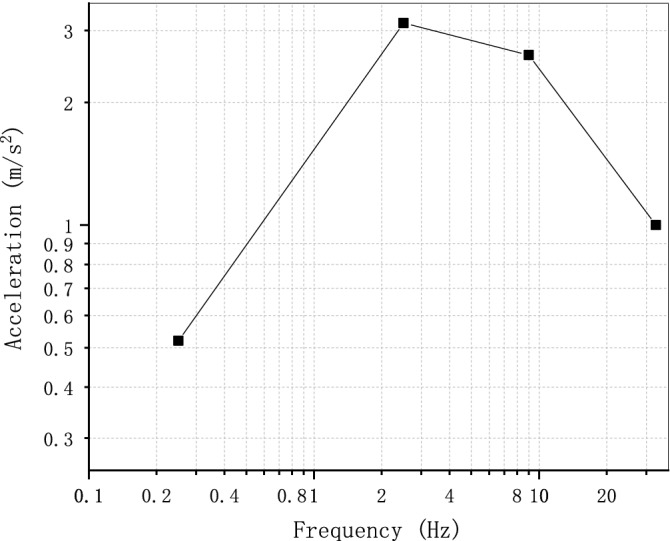
Desigen response acceleration spectrum defined by RG 1.60.

**Figure 20:**
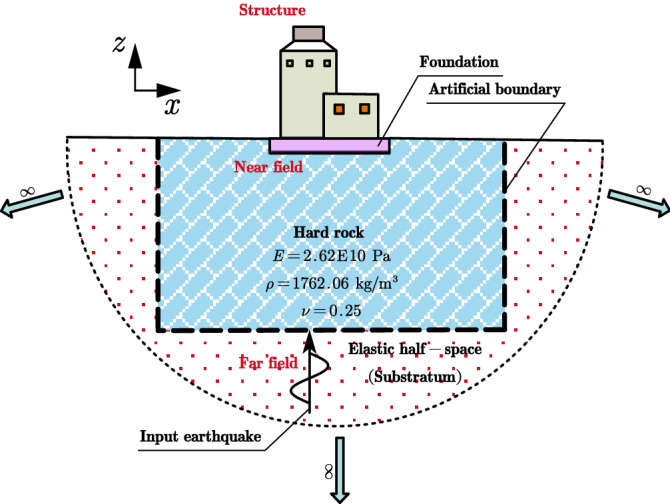
Numerical SSI model of NPP based on the direct method.

To verify the performance and robustness of the implementation that has been primarily validated in “Cantilever subjected to a harmonic excitation” section, the application in the analysis of a practical engineering problem is necessary. A typical numerical elastic SSI analysis of a NPP with direct method in the time domain is taken as an example, which includes the simulation of the structure with traditional FEM elements, near field soil with the polyhedron elements, far field soil with an artificial non-reflecting boundary condition, external excitation input with site response analysis, and interaction between them with constrain or contact. For simplicity, the SSI analysis of a NPP is elastic. But it is enough to demonstrate the merit in automatic mesh generation and ability to be integrated seamlessly with other modules of the polyhedron element based on SBFEM and its implementation by UEL. The nuclear power plant is located on elastic rock soil with the Young’s modulus of 2.63E10 Pa, Poisson’s ratio of 0.25 and density of 1762.06 kg/m^3^. Two artificial seismic plane waves vibrating along the two horizontal directions with PGA of 0.9806 m/s^2^ or 0.1 g (see Fig. 18) defined by the U.S. NRC RG 1.60^57^ response acceleration spectrum (see Fig. 19) are introduced into the SSI system from the bottom of the rock propagating upward along the vertical direction. This earthquake intensity just satisfies the minimum limit recommended by the IAEA guidelines for the SL-2 ground motion hazard level^58^. The SSI numerical analysis model of NPP based on the direct method is illustrated in Fig. 20.

#### Numerical model

The SSI numerical analysis of NPP can be divided into three individual parts: the structure, the near-field and the far-field. The structure and the near-field are modeled by FEM. The far-field is modeled by the direct method. Benefiting from the FEM for unmatched mesh based on kinematic constraints, the structure and the near-field soil can be meshed separately.

**Figure 21:**
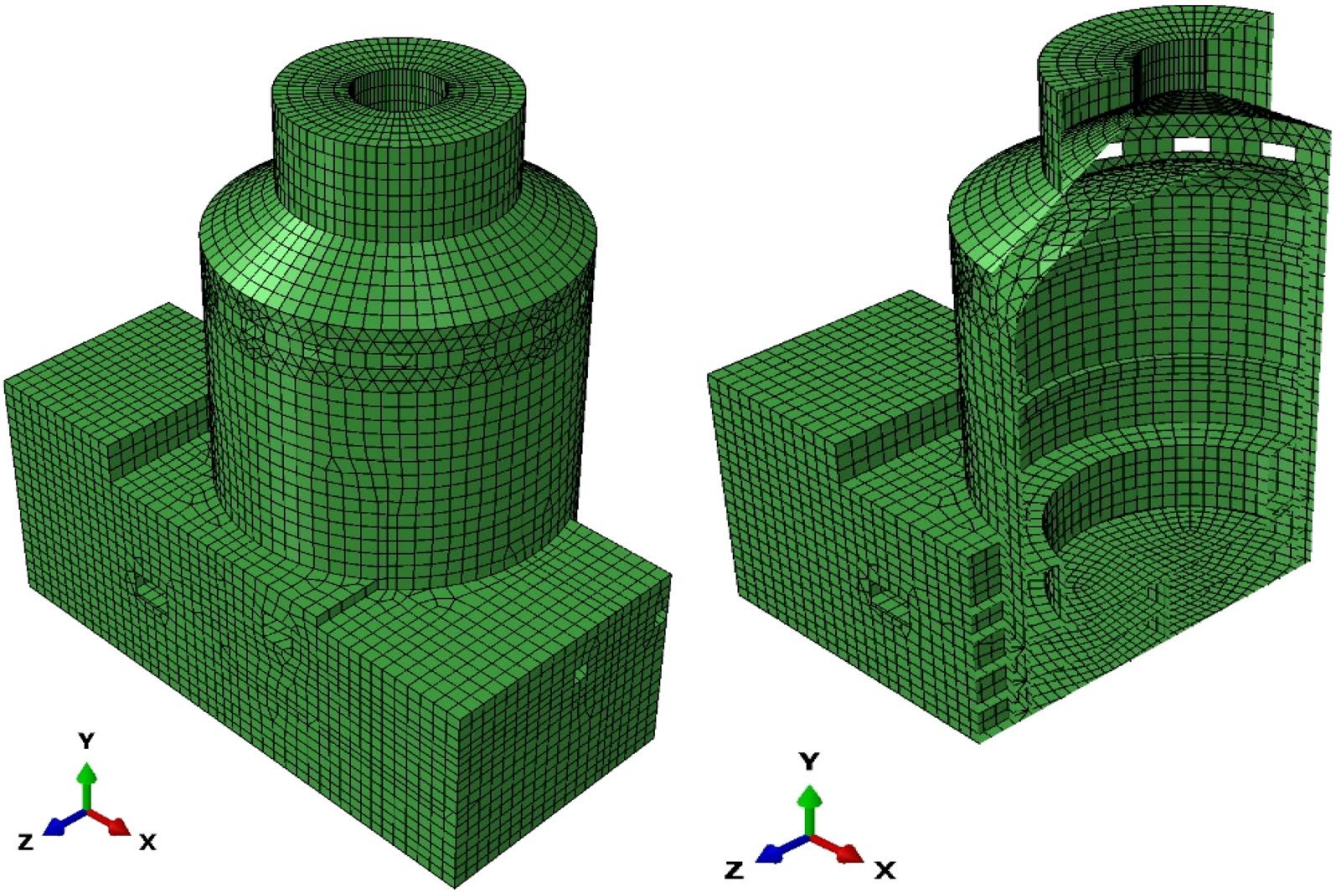
FEM model of the NPP structure.

For simplicity, the structures on the nuclear island including the containment and the auxiliary buildings are meshed with shell elements S4R or S3 (see Fig. 21). The containment of this NPP is a double shell structure including an outer reinforced concrete vessel called shield building (SB) and an inner steel vessel. The foundation of the nuclear island is meshed with solid elements embedded in the near-field soil. The connection between the structure and the near-field soil is modeled by the tie constrain defined on the interface.

**Figure 22:**
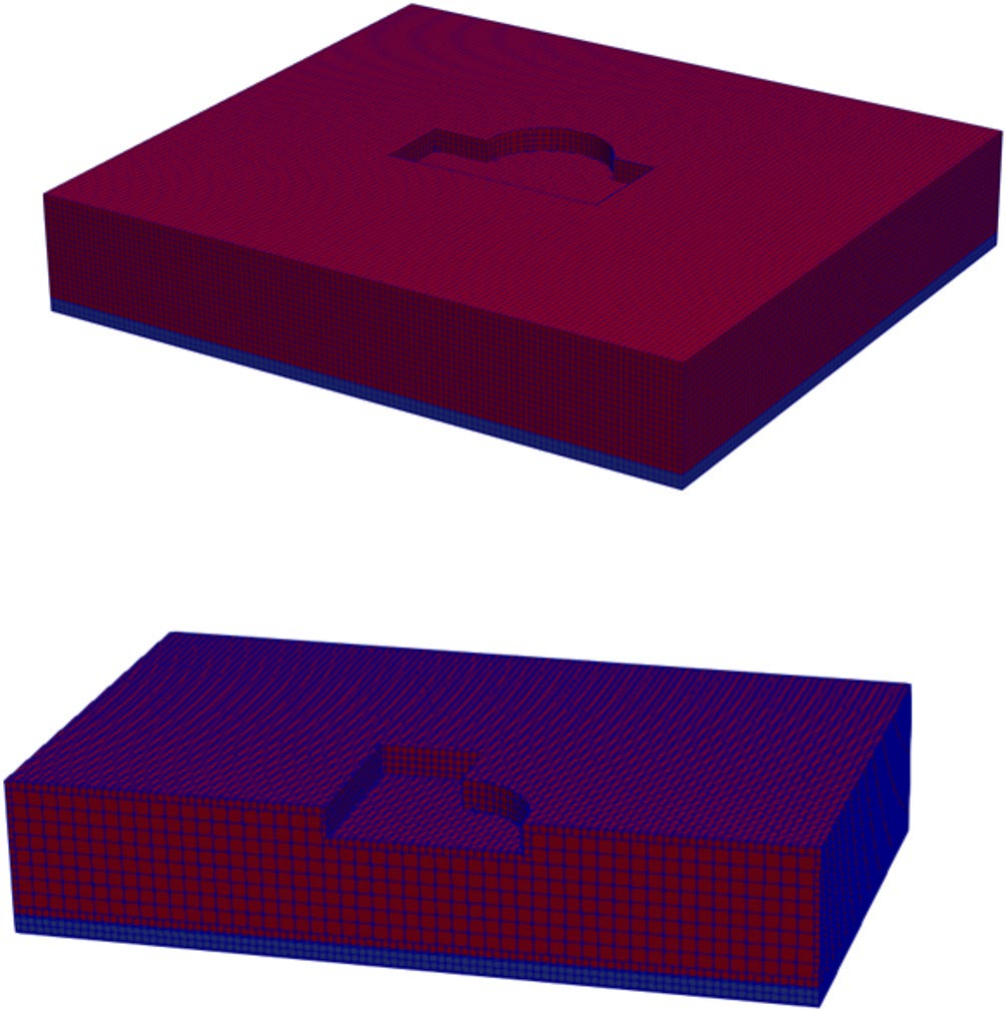
FEM model of the NPP near field with user-defined polyhedron elements.

The geometry of the near-field soil can be very complex once geological features must be considered like cracks or faults, etc. Therefore, the solid element is the most reasonable choice to model the near-field soil. Besides, in order to ensure the accuracy, the direct method requires that the size of the near-field soil should be as large as possible and no less than twice of the size of the structure consuming a lot of computing resources. Tremendous efforts for the mesh generation are needed to balance the accuracy and efficiency of the numerical model. In this example, the near-field soil is meshed with octree solid elements automatically (see Fig. 22). Although the geometry of the near-field soil in the example is rather simple, the geological complexity of the soil should not cause any difficulties in the automatic mesh generation process.

The SSI effect is considered by applying an artificial non-reflecting boundary condition (NRBC) at the truncation of the near field soil. Specifically, a set of springs and dashpots tied to the ground along the three directions are added to the nodes on the interface between the near-field and far-filed soil representing the approximation of the viscoelastic NRBC^59^. Unlike the substructure method, the corresponding input of exogenous excitations for a specific NRBC is needed. For viscoelastic boundary, the seismic load is imported by equivalent forces acting on the nodes of the artificial boundary^59,60^. The Rayleigh damping is applied to keep the damping ratio equal to 0.05 at the frequencies of 2.0 Hz and 15.0 Hz with the coefficient of $\alpha = 1.108797$ and $\beta = 0.000936$.

There are totally 260,499 elements, 167,221 nodes, and 549,177 degrees of freedom in this NPP model, the 13 section properties of which are summarized in Table 5. A transient dynamic analysis based on HHT implicit time integration is performed with a fixed time step of 0.01 s.

**Table 5:** Section properties of the SSI model of the nuclear power plant.

Section name	Thickness (m)	Density (kg/m^3^)	Young’s modulus (Pa)	Poisson’s ratio	Element type	Additional mass (kg/m^2^)	Location
S1	0.38	2300	3.35E10	0.2	S4R/S3	1136.2	Structure
S2	0.912	2300	3.35E10	0.2	S4R/S3	2728.88	Structure
S3	0.6	2300	3.35E10	0.2	S4R/S3	1794	Structure
S4	0.2286	2300	3.35E10	0.2	S4R/S3	683.514	Structure
S5	0.762	2300	3.35E10	0.2	S4R/S3	2278.38	Structure
S6	3.9768	2300	3.35E10	0.2	S4R/S3	111,889.8	Structure
S7	4.6048	2300	3.35E10	0.2	S4R/S3	13,768.3	Structure
S8	0.45	2300	3.35E10	0.2	S4R/S3	1345.5	Structure
S9	0.048	7800	2.0E11	0.3	S4R/S3	486.72	Structure
S10	0.042	7800	2.0E11	0.3	S4R/S3	418.782	Structure
S11	-	2300	3.35E10	0.2	C3D8R/C3D6	–	Structure
S12	-	1762.06	2.63E10	0.25	UEL	–	Soil

#### Results and discussion

**Figure 23:**
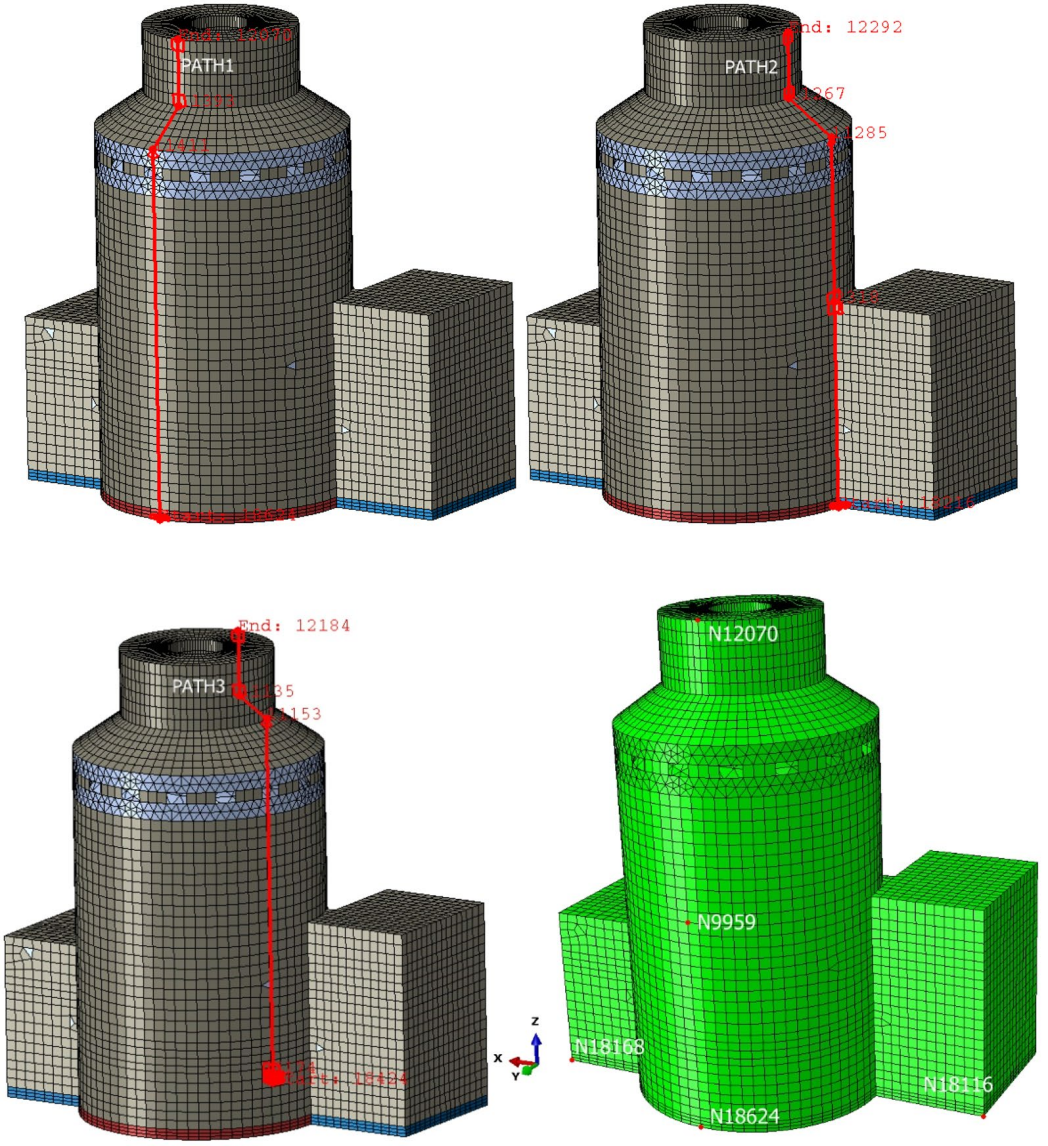
Locations of monitoring points of the nuclear power plant.

To investigate the detailed acceleration and displacement responses of the NPP, five nodes are selected as monitoring points (see Fig. 23), the locations of which are listed in Table 6.

**Table 6:** Locations of monitoring points of the nuclear power plant.

Node ID	Part	X coordinate (m)	Y coordinate (m)	Z coordinate (m)
18,624	Structure	115.5	131.355	33.576
9959	Structure	115.5	131.355	69.671
12,070	Structure	115.500	122.820	116.916
18,116	Structure	73.742	109.255	33.576
18,168	Structure	151.162	117.789	33.576

**Figure 24:**
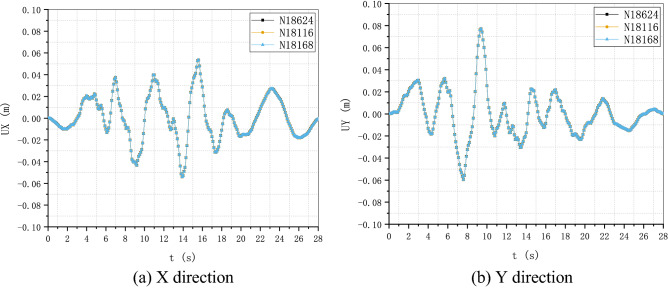
Horizontal displacement histories of monitoring points at the foundation. (**a**) X direction, (**b**) Y direction.

**Figure 25:**
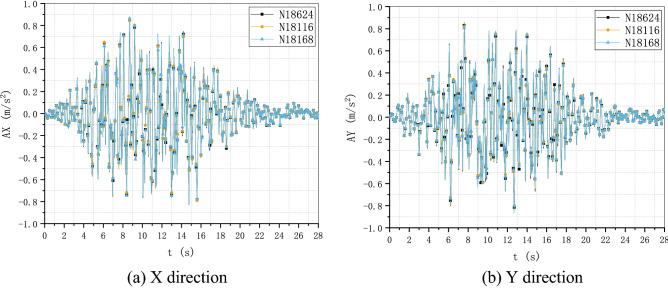
Horizontal acceleration histories of monitoring points at the foundation. (**a**) X direction, (**b**) Y direction.

**Figure 26:**
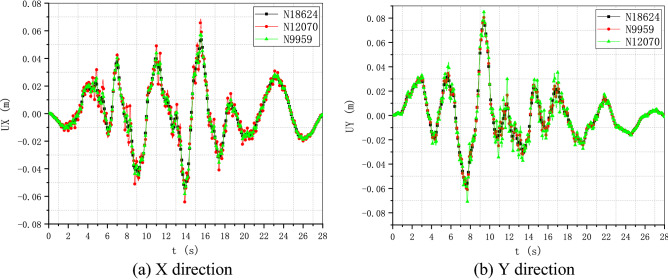
Horizontal displacement histories of monitoring points at different heights (33.576 m, 69.671 m, 116.916 m). (**a**) X direction, (**b**) Y direction.

**Figure 27:**
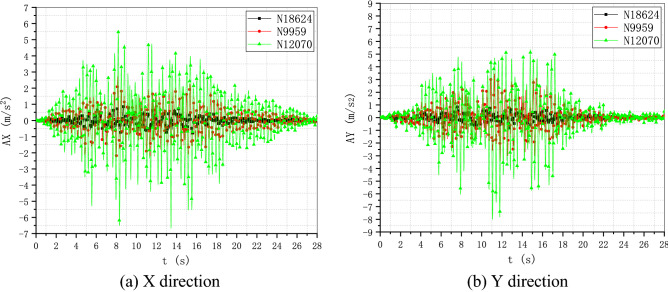
Horizontal acceleration histories of monitoring points at different heights (33.576 m, 69.671 m, 116.916 m). (**a**) X direction, (**b**) Y direction.

**Figure 28:**
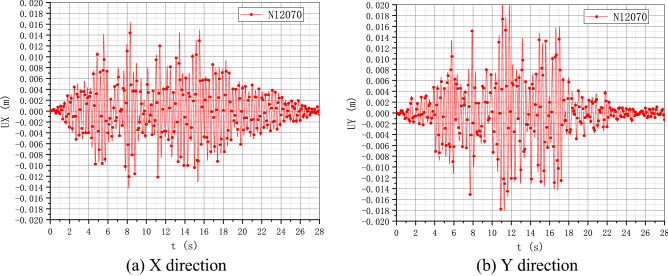
Relative horizontal displacement histories of the points on the top of the shield building. (**a**) X direction, (**b**) Y direction.

The time history of acceleration and displacement response of these nodes is compared. Figures 24 and 25 show the horizontal displacement and acceleration response of the nodes on the NPP foundation at the height of 33.576 m. It appears that the foundation is moving like a rigid plate without any rotation. The peak acceleration of the input movement at the foundation is about 0.09 g, which is a little less than the input movement at the substratum top (or the hard rock bottom). The site hardly changes the input movement due to the thin hard rock soil layer beneath the foundation indicating an insignificant SSI effect. The peak displacement of the nodes at the foundation is about 0.076 m, which can be recognized as a basic motion of the entire NPP. Figures 26 and 27 display the horizontal displacement and acceleration response of the nodes on the NPP foundation at different heights from the foundation to the top of the outer RC containment. It can be found in Fig. 26 that the peak displacement is 0.054 m (X direction) and 0.077 m (Y direction) at the height of 33.567 m, 0.058 m (X direction) and 0.081 m (Y direction) at the height of 69.671 m, 0.069 m (X direction) and 0.085 m (Y direction) at the height of 116.916 m, increasing along the vertical direction from the bottom to the top of the containment structure. In Fig. 27, the same trend that the peak accelerations increase from 0.85 m/s^2^ (X direction) and 0.81 m/s^2^ (Y direction) to 2.75 m/s^2^ (X direction) and 3.43 m/s^2^ (Y direction) to 5.47 m/s^2^ (X direction) and 7.9 m/s^2^ (Y direction) can be found. The geometry asymmetry is the main reason why there are differences in the response along the X and Y direction. The acceleration enlargement ratio from the foundation to the top is about 9.75, which is the result of the stiffness of the soil. Figure 28 illustrates the relative displacement response of the SB top to the SB foundation, eliminating the rigid transition movement due to the global site response. The peak values of the displacement in X direction and Y direction are 1.6 cm and 2.0 cm.

**Figure 29:**
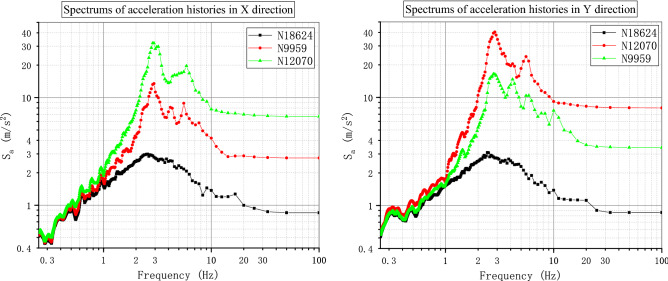
Floor response spectrums of monitoring points at different heights (33.576 m, 69.671 m, 116.916 m).

The floor response spectra of monitoring points at different heights with the damping ratio of 0.05 are listed in Fig. 29. The same trend of spectrum enlargement for the two horizontal directions can be observed. The peak spectrum values of the three nodes appear at the same frequency of 2.78 Hz, which is near the fundamental frequency of the SSI system. In the frequency range between 0.25 and 1.0 Hz, the enlargement ratio along the height is less than 1.5. The enlargement ratio increases starting from 1.0 Hz and reaches its peak value of 13.33 at 2.78 Hz. This value decreases to about 9.3 at 100.0 Hz that is nearly equivalent to the acceleration enlargement ratio from the foundation to the top of the shield building.

**Figure 30:**
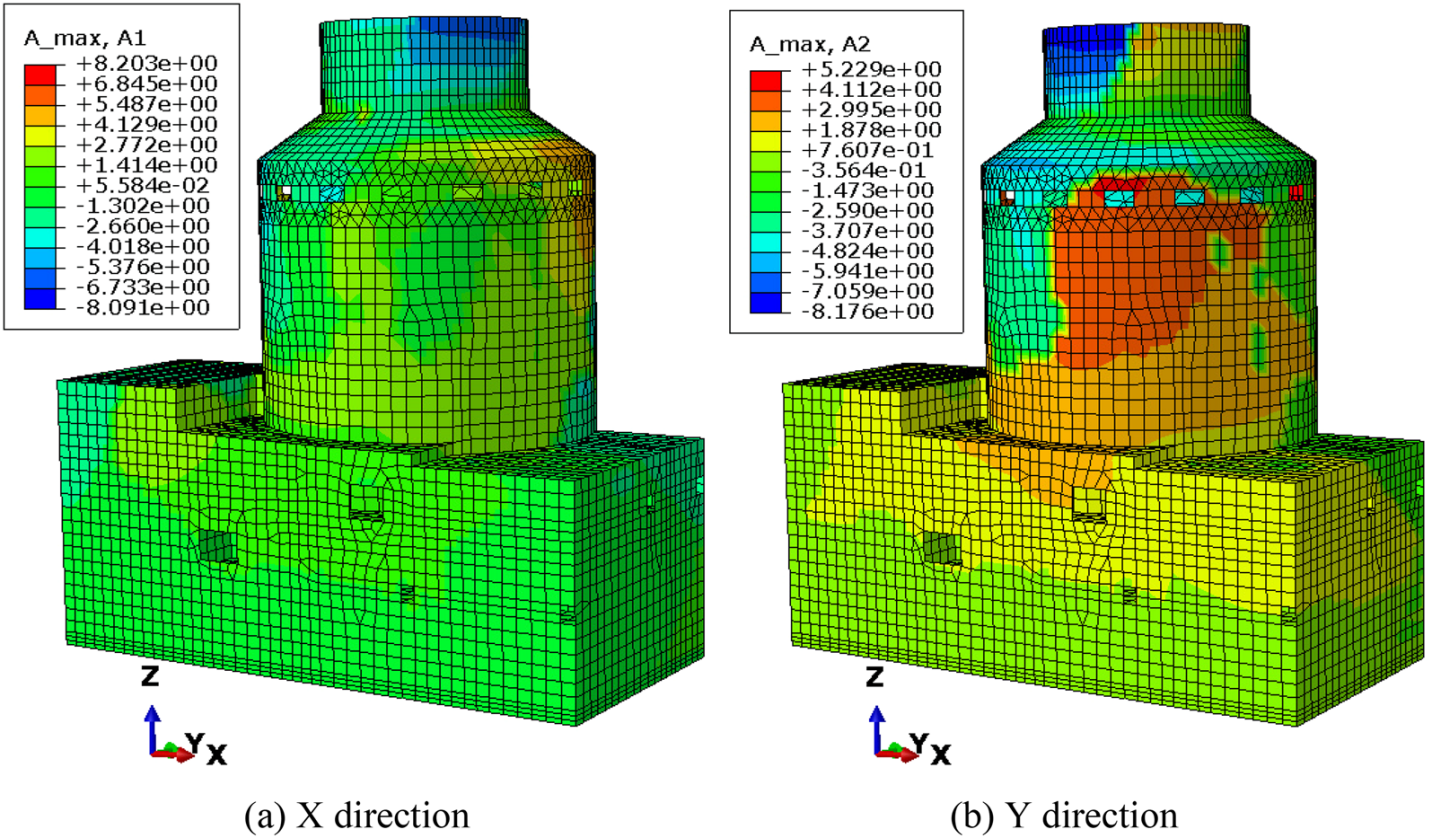
Distribution of the horizontal peak acceleration of the NPP structure. (**a**) X direction, (**b**) Y direction.

**Figure 31:**
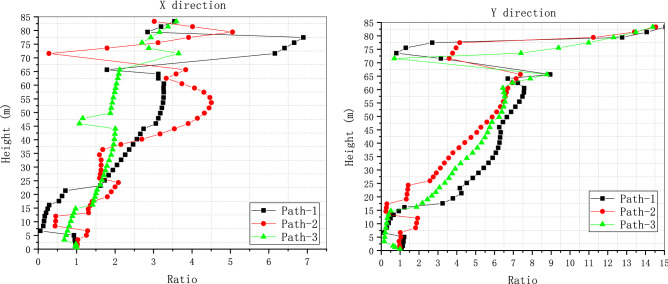
Amplification ratio of the peak acceleration along the Z direction.

The distribution of the peak acceleration (absolute values) can be seen from Fig. 30. The maximum PGA values arise at the top of the shield building. In general, the PGA values are enlarged along the vertical direction except regions near the foundation. The amplification ratio of the peak acceleration along three paths is detailed in Fig. 31. The nodes of the three paths that are parallel to Z axis are chosen as the monitoring points (see Fig. 23) and the height of the foundation bottom is set as zero. There is an upheaval of amplification ratio at the height of around 75 m where the water tank is located.

## Conclusion

The results of two examples indicate that the implementation of SBFEM-based arbitrary polyhedron elements can be adapted to the automatic FEM analysis of static and dynamic elastic problems with polyhedron meshes generated by any tool. The first example conforms the accuracy and efficiency of the polyhedron element based on SBFEM and the associated UEL implementation in ABAQUS. Through the second example, it can be found that after the geometry model of a structure is built, automatic dynamic analyses of complex or large structures can be achieved by the combination of polyhedron mesh generation technique and the implementation of arbitrary polyhedron elements in ABAQUS. On one hand, this implementation of SBFEM-based elements dispenses with the development of the pre/post-processor, matrix solver, time integration module, contact analysis module, nonlinear analysis module, etc. extending the usability of SBFEM in engineering analyses of bounded domain. On the other hand, this implementation also enhances the original function of ABAQUS to a great extent allowing engineering analyses with elements of almost any topology.

## Appendix 3: Element computation algorithm of UEL

**Table:**

Stiffness and mass matrix computation of the polyhedron element based on SBFEM
STEP 1: Prepare S-element data, including shifted coordinates of nodes and scaling center (Eq. (15)), node connectivity of each W-element, etc
STEP 2: For each W-element do:
STEP 2.1: Prepare W-element data and integration points data;
STEP 2.2: For each numerical integration point do:
STEP 2.2.1: Compute the shape functions and the their derivatives;
STEP 2.2.2: Compute the determinant of the Jacobian matrix on the boundary by Eq. (21);
STEP 2.2.3: Compute $\left[ {b^{1} } \right]$, $\left[ {b^{2} } \right]$, $\left[ {b^{3} } \right]$ by Eqs. (31)–(33);
STEP 2.2.4: Compute $\left[ {B^{1} } \right]$, $\left[ {B^{2} } \right]$ by Eqs. (45), (46);
STEP 2.2.5: Compute material property matrix $\left[ D \right]$ by Eq. (8);
STEP 2.2.6: Compute $\left[ {E^{0} } \right]$, $\left[ {E^{1} } \right]$, $\left[ {E^{2} } \right]$, and $\left[ {M^{0} } \right]$ by Eq. (57);
STEP 2.2.7: Accumulate the $\left[ {E^{0} } \right]$, $\left[ {E^{1} } \right]$, $\left[ {E^{2} } \right]$, and $\left[ {M^{0} } \right]$;
STEP 2.3: Assemble the $\left[ {E^{0} } \right]$, $\left[ {E^{1} } \right]$, $\left[ {E^{2} } \right]$, and $\left[ {M^{0} } \right]$;
STEP 3: Perform eigen-decomposition of $\left[ Z \right]$ in Eq. (62) to get sorted $\left[ \Phi \right]$ and $\left[ \lambda \right]$;
STEP 4: Compute the stiffness matrix $\left[ K \right]$ by Eq. (69);
STEP 5: Compute the mass matrix $\left[ M \right]$ by Eq. (70)–(72)
